# Beneficial effects of curtailing immune susceptibility in an Alzheimer’s disease model

**DOI:** 10.1186/s12974-019-1554-9

**Published:** 2019-08-13

**Authors:** Giulia Di Benedetto, Chiara Burgaletto, Anna R. Carta, Salvatore Saccone, Laurence Lempereur, Giovanna Mulas, Carla Loreto, Renato Bernardini, Giuseppina Cantarella

**Affiliations:** 1 Department of Biomedical and Biotechnological Sciences, Section of Pharmacology, University of Catania, Via Santa Sofia 97, 95123 Catania, Italy; 2 Department of Biomedical Sciences, University of Cagliari, Cagliari, Italy; 3 Department of Biological, Geological and Environmental Sciences, Section of Animal Biology, University of Catania, Catania, Italy; 4 Department of Biomedical and Biotechnological Sciences, Section of Anatomy & Histology, University of Catania, Catania, Italy

**Keywords:** Neurodegeneration, Immune response, Inflammation, AD target therapy

## Abstract

**Background:**

Currently, there are no effective therapeutic options for Alzheimer’s disease, the most common, multifactorial form of dementia, characterized by anomalous amyloid accumulation in the brain. Growing evidence points to neuroinflammation as a major promoter of AD. We have previously shown that the proinflammatory cytokine TNFSF10 fuels AD neuroinflammation, and that its immunoneutralization results in improved cognition in the 3xTg-AD mouse.

**Methods:**

Here, we hypothesize that inflammatory hallmarks of AD might parallel with central and peripheral immune response dysfunction. To verify such hypothesis, we used a triple transgenic mouse model of AD. 3xTg-AD mice were treated for 12 months with an anti-TNFSF10 antibody, and thereafter immune/inflammatory markers including COX2, iNOS, IL-1β and TNF-α, CD3, GITR, and FoxP3 (markers of regulatory T cells) were measured in the spleen as well as in the hippocampus.

**Results:**

Spleens displayed accumulation of amyloid-β_1–42_ (Aβ_1-42_), as well as high expression of Treg cell markers FoxP3 and GITR, in parallel with the increased levels of inflammatory markers COX2, iNOS, IL-1β and TNF-α, and blunted IL-10 expression. Moreover, CD3 expression was increased in the hippocampus, consistently with FoxP3 and GITR. After chronic treatment of 3xTg-AD mice with an anti-TNFSF10 antibody, splenic FoxP3, GITR, and the above-mentioned inflammatory markers expression was restored to basal levels, while expression of IL-10 was increased. A similar picture was observed in the hippocampus. Such improvement of peripheral and CNS inflammatory/immune response was associated with decreased microglial activity in terms of TNFα production, as well as decreased expression of both amyloid and phosphorylated tau protein in the hippocampus of treated 3xTg-AD mice. Interestingly, we also reported an increased expression of both CD3 and FoxP3, in sections from human AD brain.

**Conclusions:**

We suggest that neuroinflammation in the brain of 3xTg-AD mice triggered by TNFSF10 might result in a more general overshooting of the immune response. Treatment with an anti-TNFSF10 antibody blunted inflammatory processes both in the spleen and hippocampus. These data confirm the detrimental role of TNFSF10 in neurodegeneration, and corroborate the hypothesis of the anti-TNFSF10 strategy as a potential treatment to improve outcomes in AD.

**Electronic supplementary material:**

The online version of this article (10.1186/s12974-019-1554-9) contains supplementary material, which is available to authorized users.

## Background

Alzheimer’s disease (AD) is the most common form of dementia [1] and is characterized, besides a high burden of disease, by substantial unmet need [2]. In fact, despite the considerable number of attempts of introducing innovative neuroprotective treatments, such need remains widely unsatisfied.

Although evidence shows that the anomalous protein amyloid beta (Aβ), which exceedingly accumulates in the hippocampus and other cerebral areas of the Alzheimer’s brain, plays a pivotal role in the pathogenesis of the disease [3, 4], outcomes of Aβ-based clinical trials have been, so far, deluding [5].

On the other hand, in the past two decades, laboratory research has shed more light on various AD-related candidate pathogenetic factors, including superoxides [6, 7], excitotoxicity [8, 9], as well as inflammation [10, 11]. In this line, peripheral immune cells have more recently been indicated as factors disrupting the immune equilibrium of the brain, potentially contributing to neurodegeneration [12].

Consistently, TNF-related apoptosis inducing ligand (TNFSF10), a potent pro-apoptotic member of the TNF superfamily also produced by injured neurons [13] and activated glia [14], is known to modulate cell-mediated immune response [15], for example, by inducing an increase of the Treg cell subset of T lymphocytes [16], and it is in fact regarded as a critical regulator of autoimmune T cells [17]. While there is evidence that the blockade of TNFSF10 improves pathology in models of multiple sclerosis, beneficial effects in other neurological pathologies, included AD, remain to be investigated [18].

In the AD brain, TNFSF10 sets into motion the inflammatory machinery, and mediates Aβ-induced neuronal death [19]. In fact, the role of TNFSF10 in Aβ-related neurotoxicity has been demonstrated by different studies showing that neutralization of TNFSF10 death pathway protects human neuronal cell line from beta-amyloid neurotoxicity [13], and, that the blockade of the TNFSF10-death receptor DR5 with a specific antibody completely prevents Aβ-induced neurotoxicity in neuronal cells in vitro [20]. Moreover, TNFSF-10 immunoreactivity has been detected in the vicinity of Aβ plaques in post-mortem human AD brains [21]. Consistently, age-related, Aβ-dependent progression of cognitive decline is efficiently prevented by chronic anti-TNFSF10 treatment in vivo in the 3xTg-AD, a triple transgenic mouse model of Alzheimer’s disease [19].

Such prominent neurotoxic effect of TNFSF10 relies upon its capacity of redundantly recruiting other inflammatory cytokines, so to synergistically contribute to the worsening of neuronal function [22].

Despite poor evidence is currently available regarding the influence of peripheral immune response upon the pathology of the AD brain and the related clinical outcome [23, 24], more recent data indicate in fact that not only peripheral immunocytes can enter the brain in murine models of AD but also their modulation significantly influence progression of brain pathology in the same animals [12].

With such rationale, and with the aim to better highlight a hypothetical prominent role of the peripheral immune system in AD, we here investigate whether immunoneutralization of TNFSF10 is related to a re-balance of both the central and the peripheral immune response, and whether this could correlate with decreased Aβ burden in the CNS, consistently with the previously demonstrated improvement of the cognitive outcome in 3xTg-AD mice [19].

To accomplish this task, we used a triple transgenic mouse model of AD (3xTg-AD), a strain homozygous for the *Psen1* mutation and homozygous for the co-injected *APPSwe* and *tauP301L* transgenes, which presents an age-dependent increase of Aβ oligomer accumulation, extracellular plaques in the cortex and the hippocampus, and tau pathology paralleled by learning and memory impairment [25]. Mice were treated chronically, twice a month for 12 months with intraperitoneal injection of an anti-TNFSF10 monoclonal antibody as previously described [19], to evaluate the effects of TNFSF10 neutralization, upon parameters of neuroinflammation, as well as upon those related to the systemic immune response.

## Methods

### Animals

Male 3xTg-AD mice [B6129-Psen1tm1MpmTg (APPSwe, tauP30L) 1Lfa/J] [25] and wild-type mice (B6129SF2/J) were purchased from Jackson Laboratories. The 3xTg-AD, overexpressing mutant APP (APPSwe), PSEN1 (PS1M146V), and hyperphosphorylated tau (tauP301L), were originally generated by co-injecting two independent transgene constructs encoding human APPSwe and tauP301L (4R/0 N) (controlled by murine Thy1.2 regulatory elements) in single-cell embryos harvested from mutant homozygous PS1M146V knock-in mice. Wild-type mice of mixed genetic background 129/C57BL6 were used as controls. These mice have been characterized and described by Oddo et al. [25]. The animals were maintained on a 12-h light/dark cycle in temperature- and humidity-controlled rooms, and food and water were available ad libitum. All experiments were carried out according to the Directive 2010/63/EU and the Italian law (D.Lgs. 26/2014), and were approved by the Italian Ministry of Health.

### Drug administration and experimental groups

Twenty 3xTg-AD and 20 wild-type mice were enrolled at 3 months of age and four study groups were used: (i) wild-type plus vehicle (Purified Rat IgG2ακ Isotype Control; BD Biosciences, San Jose, CA, USA); (ii) wild-type plus TNFSF10-neutralizing antibody (Purified Rat Anti-Mouse CD253;BD Biosciences); (iii) 3xTg-AD plus vehicle; and (iv) 3xTg-AD plus TNFSF10-neutralizing antibody. Animals (ten per experimental group) were administered with TNFSF10-neutralizing antibody (concentration: 0.05 mg/ml; 200 μl/ mouse; i.p.) or vehicle (concentration: 0.05 mg/ml; 200 μl/ mouse; i.p.) twice a month (Monday at 12 a.m.) and sacrificed at 15 months of age 2-weeks after the last injection.

### Human brain samples

Hippocampus tissue slides were obtained from four healthy donors per group (two males, two females, age 59–85 years; post-mortem delay 5–8 h, hippocampal CA2-CA3 subfields) who had no known history of neurological or psychiatric disease. Human AD samples were obtained from patients (two males, two females; age 65–80 years; post-mortem delay 4–6 h; hippocampal CA2-CA3 subfields). Tissues were purchased from Abcam (Cambridge, UK).

### Immunohistochemistry

For immunohistochemical analysis, sections were incubated for 30 min in 0.3% H_2_O_2_/methanol to quench endogenous peroxidase activity, then rinsed for 20 min with phosphate-buffered saline (PBS; Bio-Optica, Italy). High-temperature antigen unmasking was conducted using a microwave oven.

Then, the sections were incubated with diluted rabbit anti-beta-amyloid 1–42 (Merck Millipore, Darmstadt, Germany). After overnight incubation in a humidified chamber (4 °C), sections were incubated with the secondary antibody (for 30 min at RT); detection was performed with the Streptavidin-biotin method using 3,3′-diaminobenzidine (DAB) as chromogen (LSAB 2 System-HRP, Dako, Denmark). Sections were counterstained with hematoxylin (Histolab Products AB, Goteborg, Sweden) mounted in GVA mount (Zymed, Laboratories Inc., San Francisco, CA, USA) and observed under an Axioplan (Zeiss, Germany) light microscope and photographed with a digital camera (Canon, Japan). The antibodies-staining (beta-amyloid 1–42) status was identified as either negative or positive. Immunohistochemical positive staining was defined by the presence of brown chromogen on the edge of the hematoxylin-stained cell nucleus, distributed within the cytoplasm or in the membrane via evaluation with light microscope. Five fields of each sample, randomly selected from each section, were analyzed for morphometric and densitometric analysis. The percentage areas (morphometric analysis) stained with antibodies (anti-beta-amyloid 1–42), expressed as % positive, dark brown pixels of the analyzed fields, and the level (high/low) of staining intensity of positive areas (densitometric analysis), expressed as densitometric count (pixel2) of positive, dark brown pixels of the analyzed fields, were calculated using an image acquisition software (AxioVision Release 4.8.2—SP2 Software, Carl Zeiss Microscopy GmbH, Jena, Germany). Digital micrographs were taken using the Zeiss Axioplan light microscope (Carl Zeiss, Oberkochen, Germany) fitted with a digital camera (AxioCam MRc5, Carl Zeiss, Oberkochen, Germany).

### Protein extraction

Tissues were lysed in a lysis buffer containing 150 mM NaCl, 50 mM Tris–HCl (pH 7.5), 5 mM EDTA, 1 mM Na_3_VO_4_, 30 mM sodium pyrophosphate, 50 mM NaF, 1 mM acid phenyl-methyl-sulphonyl-fluoride, 5 μg/ml aprotinin, 2 μg/ml leupeptin, 1 μg/ml pepstatin, 10% glycerol, and 0.2% TritonTM X-100. The homogenates were then centrifuged at 14,000 rpm for 10 min at 4 °C. The protein concentration of the supernatant was determined by the Bradford method [26].

### Western blot analysis

Equal amounts of protein (50 μg) were resolved by 8–12% SDS-PAGE gels and transferred onto Hybond-ECL nitrocellulose membranes (GE Healthcare, Little Chalfont, UK). Membranes were blocked at room temperature overnight with a blocking solution composed of 5% nonfat dry milk (Bio-Rad Laboratories, Segrate, Italy) in phosphate-buffered saline plus 0.1% Tween 20 (PBS-T) and were then probed with the following appropriate primary antibodies: rabbit anti-beta-amyloid1–42 antibody (Merck Millipore, Darmstadt, Germany); rabbit anti-TNFRSF10B (Abcam); rabbit anti-TNFSF10 (Abcam); goat anti-GITR (R&D Systems, Inc.); rat anti-FoxP3 (eBioscience, San Diego, CA, USA); rabbit anti-IL10 antibody (Abbiotec, San Diego, CA, USA); mouse anti-COX2 (BD Biosciences); rabbit anti-NOS2 (Santa Cruz Biotechnology Inc., Santa Cruz, CA, USA); goat anti-IL-1β (R&D Systems, Inc.); and rabbit anti-TNF-α antibody (Abbiotec). After that, the membranes were washed with PBS-T, and finally were probed with the appropriate horseradish peroxidase-conjugated secondary antibodies (GE Healthcare) for 60 min at room temperature in 5% non-fat dry milk (full details of the antibodies used are reported in Additional file 8: Table S1). For immunodetection, the membranes were exposed to film after enhanced chemiluminescence (ECL) (GE Healthcare). β-actin and β-tubulin (Santa Cruz Biotechnology Inc.) were used as an internal control to validate the right amount of protein loaded on the gels. Densitometric analysis of band intensity was performed with the aid of ImageJ software (https://imagej.nih.gov/ij/).

### Immunofluorescence and confocal microscopy analysis

Splenic and hippocampal tissue specimens were fixed overnight in 10% neutral-buffered formalin (Bio-Optica). After overnight washing, they were dehydrated in graded ethanol and paraffin-embedded taking care to preserve their anatomical orientation. Tissue sections of 5 mm were then cut and mounted on silanized glass slides and air dried. To remove the paraffin, slides were immersed in xylene two times, for 3 min each; rehydrated with graded ethanol, 100%, 95%, 80%, 70%, and 50%, for 3 min each; and transferred to tap water. Antigens were retrieved in sodium citrate buffer (10 mM sodium citrate, 0.05% Tween-20, pH 6.0) by microwave for 15 min, followed by rinsing with tap water. The slides were then washed in PBS containing 0.25% Triton X-100 (PBST) twice for 5 min each, blocked in PBST/1% BSA for 1 h at room temperature, briefly rinsed with PBST, and incubated at 4 °C overnight with the following primary antibodies: a rabbit anti-CD3 antibody (Abcam), or a rat anti-FoxP3 antibody (eBioscience), or a mouse anti-GITR antibody (Santa Cruz Biotechnology Inc.), or a rabbit anti-IL10 antibody (Abbiotec), or a rabbit anti-beta-amyloid1–42 antibody (Merck Millipore), or a goat anti-p-TAU antibody (Santa Cruz Biotechnology Inc.), or a rat anti-CD11b antibody (Serotec, Kidlington, UK), or a rabbit anti-TNF-α antibody (Abbiotec). Antibodies were applied directly onto sections before overnight slide incubation (4 °C) in a humid chamber. For fluorescence visualization, after washing in PBS three times for 5 min each, sections were incubated with the corresponding fluorescent-labeled secondary antibodies at dark for 1 h at room temperature: goat anti-mouse IgG-TR (Santa Cruz Biotechnology Inc.); or goat anti-rat IgG antibody, FITC conjugate (Merck Millipore); or goat anti-rabbit IgG-TR (Santa Cruz Biotechnology Inc.); or Alexa Fluor 488 goat anti-rabbit IgG (Life Technologies, Carlsbad, CA, USA); or Alexa Fluor 488 donkey anti-goat (Life Technologies). See Additional file 8: Table S1 for full details of the antibodies used. Finally, for staining of nuclei and stabilization of fluorescent signals, slides were covered in mounting medium (Fluoroshield with DAPI; Sigma-Aldrich, Milan, Italy) and secured with a coverslip. Images were observed using a laser scanning confocal microscope (Zeiss LSM 700, Germany).

Qualitative and quantitative analysis for CD11b and TNF-α immunoreactivity was performed using a Leica 4D confocal laser scanning microscope, equipped with an argon–krypton laser. Confocal images were generated and processed as described [27]. Volume of co-localized elements was determined by Imaris 7.3 as previously described [27]. Four regions of interest (*x* = 40 μm; *y* = 40 μm; *z* = 10 μm) were randomly chosen within each slide and their respective volumes were calculated, summed, and expressed as volume/μm^3^ [28].

### Statistical analysis

Data were analyzed either by the one-way or the two-way analysis of variance (ANOVA), followed, respectively, by the Duncan’s least significant difference test or by Bonferroni post-hoc test. Vertical bars are means ± S.E.M. of at least three different experiments; significance was set at a *p* value < 0.05 or *p* < 0.001. The graphs were made using Graph Pad Prism (Ver. 7, La Jolla, USA). Statistical evaluation was performed using standard computer software (SPSS software package, ver. 23.0, SPSS Inc., Chicago, IL, USA).

## Results

### Chronic anti-TNFSF10 treatment beneficially influences body, spleen, and brain weight and decreases the amount of amyloid beta in the spleen of 3xTg-AD mice

**Fig. 1:**
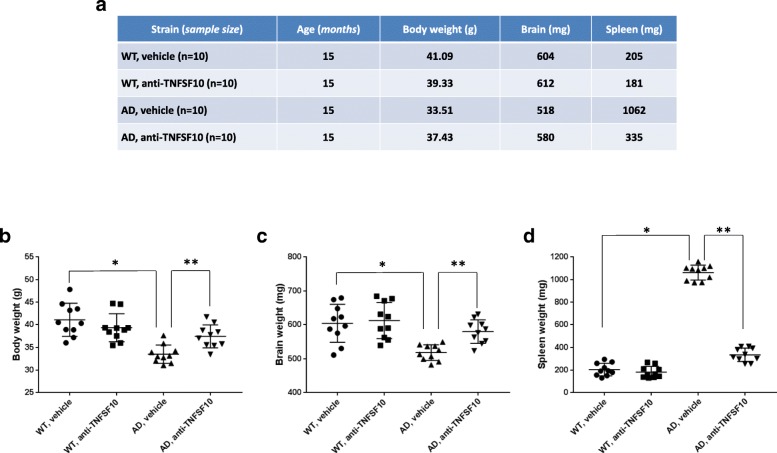
Panel **a** (table): body, brain, and spleen weight changes in 15-month-old 3xTg-AD mice treated for 12 months with an anti-TNFSF10 monoclonal antibody (10 μg, i.p. twice a month). Panels **b**–**d**: comparison of body, brain and spleen weights in the same animals. *p < 0.05 vs WT treated with vehicle; **p < 0.05 vs. untreated 3xTg-AD mice (one-way ANOVA, followed by a Duncan’s multiple range test). Vertical bars are means ± S.E.M. *WT* wild-type (*n* = 10/group); AD: 3xTg-AD mice (*n* = 10/group)

In light of the reported differences in the size of 3xTg-AD animals compared to wild-type [29], we measured the weight of the body, brains, and spleens, confirming that while both body and brain weight were significantly lower in 3xTg-AD mice vs. wild-type animals, spleens weight was significantly augmented (Fig. 1a). Treatment with the anti-TNFSF10 antibody resulted in increased body and brain weight in 3xTg-AD animals and, on the other hand, in a significant reduction of the spleen weight (Fig. 1a–d).

**Fig. 2:**
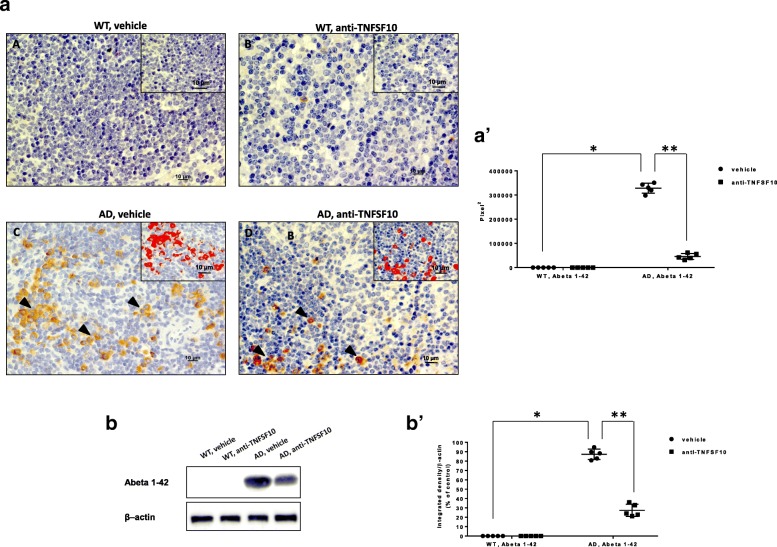
Panel (a): representative pictures of the immunohistochemical detection of Aβ1–42 expression (black arrows), in the spleen of 3xTg-AD treated for 12 months with an anti-TNFSF10 monoclonal antibody (10 μg, i.p. twice a month). Photos A and B: respectively, wild-type mice untreated, or treated with the anti-TNFSF10 antibody. Photos C and D: respectively 3xTg-AD mice untreated or treated with the anti-TNFSF10 antibody. The inserts in photos represent the respective areas magnified. Scale bar = 10 μM. Panel (a’): densitometric count of Aβ1–42 immunopositive cells. *p < 0.05 3xTg-AD mice untreated vs WT treated with vehicle; **p < 0.05 3xTg-AD mice treated with anti-TNFSF10 vs. untreated 3xTg-AD mice (one-way ANOVA, followed by a Duncan’s multiple range test). Vertical bars are means ± S.E.M. *WT* wild-type (*n* = 5/group); AD: 3xTg-AD mice (*n* = 5/group). Panel (b): Western blot analysis of Aβ1–42 in splenic homogenates. Panel (b’): Densitometric analysis of the representative Western blot *p < 0.05 3xTg-AD mice untreated vs WT treated with vehicle; **p < 0.05 3xTg-AD mice treated with anti-TNFSF10 vs. untreated 3xTg-AD mice (one-way ANOVA, followed by a Duncan’s multiple range test). Vertical bars are means ± S.E.M. *WT* wild-type (*n* = 5/group); AD: 3xTg-AD mice (*n* = 5/group)

Immunohistochemical and Western blot analysis performed on spleen samples revealed specific immunoreactivity for amyloid beta 1–42 in AD vehicle (Fig. 2(a, C, black arrows; b)) and AD anti-TNFSF10 (Fig. 2(a, D, black arrows; b)) spleen sections, while spleens from WT animals (untreated; treated with anti-TNFS10) did not show any specific immunoreactivity for amyloid beta 1–42 (Fig. 2(a, A and B, respectively; b)).

Densitometric analysis showed that the expression of amyloid beta 1–42 was significantly higher in untreated AD animals compared to anti-TNFSF10 treated AD animals (Fig. 2(a’, b’)).

### Effects of chronic anti-TNFSF10 treatment on the expression of either proinflammatory and anti-inflammatory molecules in the spleen of 3xTg-AD mice

To corroborate the hypothesis that such changes in 3xTg-AD mice spleen could be related with impairment of some proinflammatory parameters, Western blot analysis was performed on spleen homogenates for the expression of TNFSF10 and its receptor TNFRSF10B, and that of the inflammatory molecules COX2, iNOS, IL-1β, TNF-α, the anti-inflammatory cytokine IL-10, as well as that of the Treg lymphocyte markers GITR and FoxP3.

**Fig. 3:**
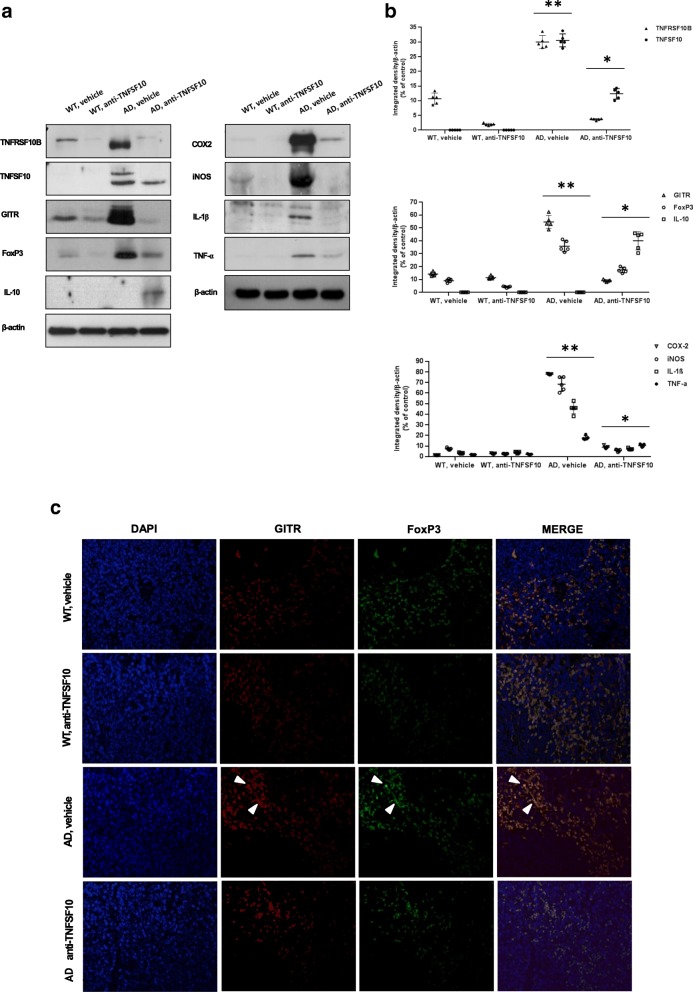
Panel **a**: effects of the treatment with an anti-TNFSF10 monoclonal antibody on the expression of either TNFSF10, TNFRSF10B receptor, proinflammatory, and anti-inflammatory molecules in the spleen of 3xTg-AD mice. *Left blots:* changes in the expression of TNFSF10 and its TNFRSF10B receptor, as well as in GITR and Foxp3 and the anti-inflammatory cytokine IL-10 expression in 3xTg-AD mice, following chronic treatment (12 months) with an anti-TNFSF10 monoclonal antibody (10 μg/animal twice a month, i.p.) or vehicle (10 μg/animal twice a month, i.p.). *Right blots:* changes in the expression of the proinflammatory mediators COX2, iNOS, IL-1β, TNF-α in 3xTg-AD mice, following chronic treatment (12 months) with an anti-TNFSF10 monoclonal antibody or vehicle (10 μg/animal twice a month, i.p.). Panel **b**: densitometric analysis of respective western blots. *p < 0.05 vs untreated 3xTg-AD mice; **p < 0.05 vs. all other matching groups (one-way ANOVA, followed by a Duncan’s multiple range test). Vertical bars are means ± S.E.M. Panel **c**: representative immunofluorescence photographs of mice spleens for GITR (red) and FoxP3 (green) expression and co-localization of the two molecules (white arrows; merge column; DAPI = nuclear staining) in WT and 3xTg-AD animals receiving either vehicle or an anti-TNFSF10 monoclonal antibody (10 μg/animal twice a month, i.p.) for 12 months. *WT* wild-type mice (*n* = 5/group); AD: 3xTg-AD mice (*n* = 5/group)

The expression of COX2, iNOS, IL-1β, and TNF-α was substantially increased in untreated 3xTg-AD mice while treatment with the anti-TNFSF10 monoclonal antibody determined a significant attenuation of their expression. On the other hand, the expression of IL-10 was significantly increased in anti-TNFSF10-treated animals. Furthermore, the expression of GITR and Foxp3, constitutively highly expressed in the spleen of untreated 3xTg-AD mice, was significantly attenuated in animals undergone the anti-TNFSF10 treatment, as confirmed by densitometric analysis (Fig. 3a, b).

In order to investigate the relationship between the variation in the expression of both GITR and FoxP3, we performed immunofluorescence of splenic sections from the same animals. Indeed, immunofluorescence confirmed that the expression of the two molecules was higher in untreated 3xTg-AD mice and it was decreased after treatment with the anti-TNFSF10 antibody. Interestingly, GITR and FoxP3 co-localized within the same cells (Fig. 3c, white arrows).

### The neuroinflammatory hallmarks in the hippocampus of 3xTg-AD mice are consistent with the splenic inflammatory pattern and are modulated by the anti-TNFSF10 treatment

**Fig. 4:**
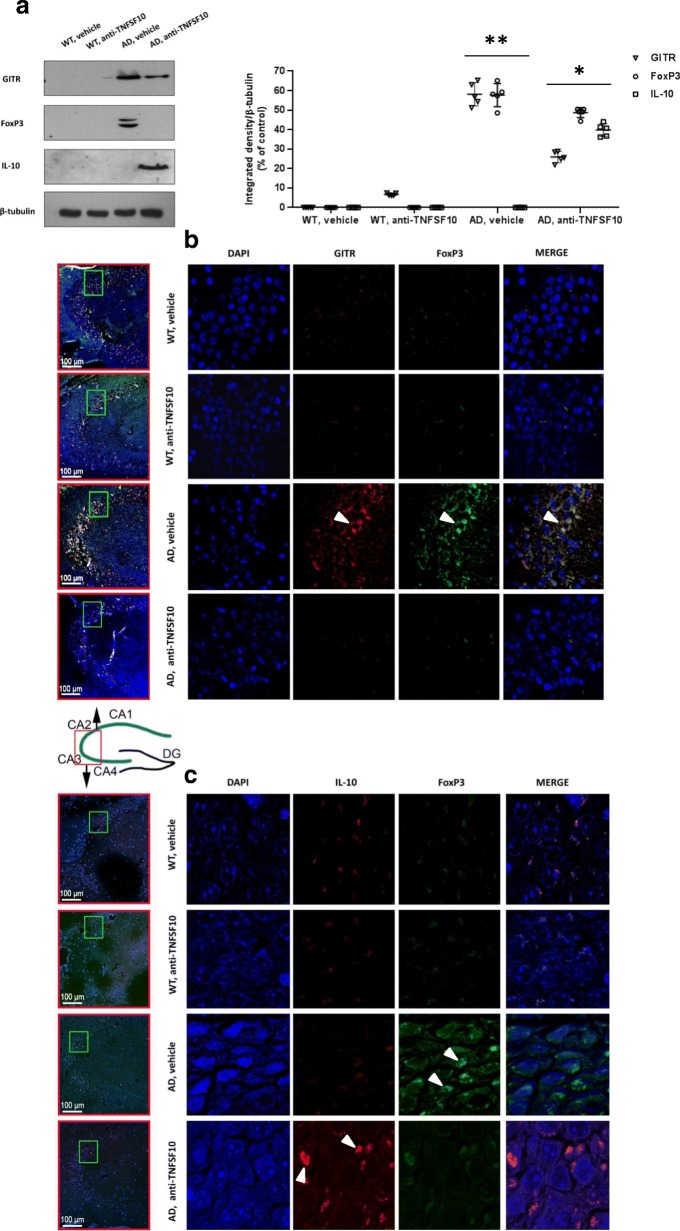
Effects of the treatment with an anti-TNFSF10 monoclonal antibody on the expression of GITR and FoxP3, as well as of the anti-inflammatory protein IL-10 in the hippocampus of 3xTg-AD mice Panel **a**: *Left*: Western blot analysis of GITR, FoxP3, and IL-10 protein expression in the mice following chronic treatment (12 months) with an anti-TNFSF10 monoclonal antibody (10 μg/animal twice a month, i.p.) or vehicle (10 μg/animal twice a month, i.p.). *Right*: Densitometric analysis of Western blots. *p < 0.05 vs untreated 3xTg-AD mice; **p < 0.05 vs. all other matching groups (one-way ANOVA, followed by a Duncan’s multiple range test). Vertical bars are means ± S.E.M. Panel **b**: representative immunofluorescence images of hippocampi for GITR (red) and FoxP3 (green) expression and co-localization from the same animal groups as above (white arrows; merge column; DAPI = nuclear staining). Panel **c**: immunofluorescence by confocal microscopy of hippocampi for IL-10 (red; white arrows) and FoxP3 (green; white arrows) expression and co-localization from the same animal groups as above (merge column; DAPI = nuclear staining). *WT* wild-type animals (*n* = 5/group); AD: 3xTg-AD animals (*n* = 5/group); the respective side columns are the lower magnification samples where the areas to analyze (CA1, CA2, CA3, and CA4) were magnified (framed in a green box)

In light of the observed splenic alterations, which reflected objective impairment of the peripheral immune response, we further investigated whether the spleen findings could relate with the neuroinflammatory processes in the hippocampus of the same animals. Thus, we studied Treg cells profile markers by means of Western blot analysis (and respective densitometry) in the hippocampus. Robust expression of GITR and FoxP3 was detectable in the brain of untreated AD mice, while treatment with the anti-TNFSF10 antibody resulted in blunted expression of both GITR and FoxP3 (Fig. 4a).

In contrast, Western blot analysis (and relative densitometry) showed that, while the expression of the anti-inflammatory cytokine IL-10 was absent in the hippocampus of 3xTg-AD mice, it became detectable in animals undergone the anti-TNFSF10 treatment (Fig. 4a).

Consistently, additional immunofluorescence experiments demonstrated that, in the same hippocampal areas, FoxP3 was abundantly expressed and co-localized with GITR immunoreactivity in untreated 3xTg-AD mice (Fig. 4b; white arrows). Treatment with the anti-TNFSF10 antibody significantly reduced the expression of both FoxP3 and GITR in the hippocampi of 3xTg-AD mice (Fig. 4b). See Additional file 1: Figure S1 for negative controls.

Moreover, as the observed immune processes were blunted by the anti TNFSF10 treatment, it was of interest that the expression of IL-10 was increased in 3xTg-AD mice treated with the anti TNFSF10 antibody, suggesting a setting into motion of the anti-inflammatory response in the brain of the 3xTg-AD animals, while FoxP3 expression was downregulated (Fig. 4c; white arrows). See Additional file 2: Figure S2 for negative controls.

**Fig. 5:**
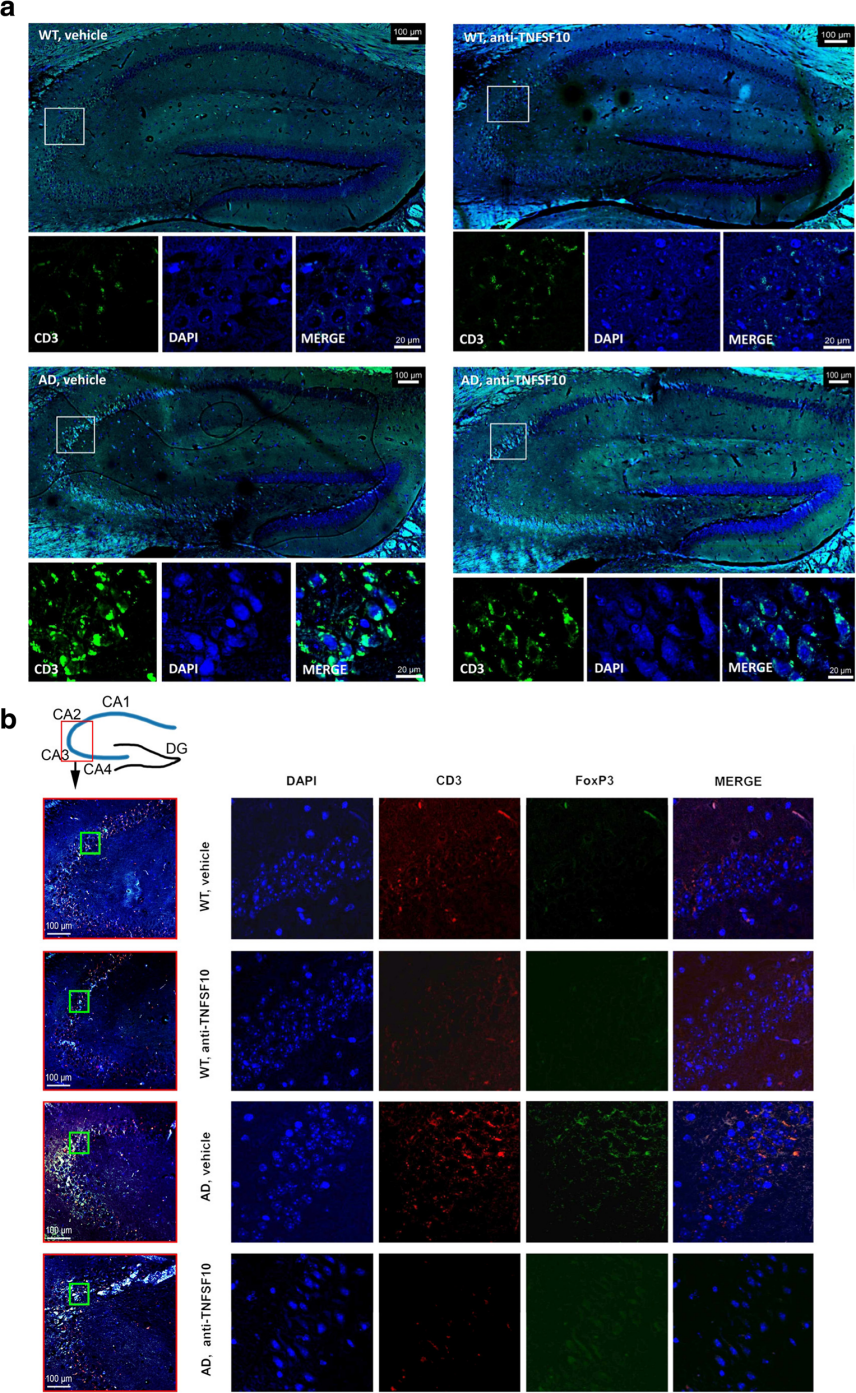
Confocal microscopy for detection of CD3 and FoxP3 positive cells in the hippocampus of 3xTg AD mice, following chronic treatment (12 months) with an anti-TNFSF10 monoclonal antibody (10 μg/animal twice a month, i.p.) or vehicle (10 μg/animal twice a month, i.p.). Panel **a**: each picture represents a single group of treatment and illustrates either the whole hippocampus (sagittal section) or, below, magnification of CA2-CA3 areas displaying specific CD3 (green) immunofluorescence (magnifications of respective white frames) in the mice following chronic treatment (12 months) with an anti-TNFSF10 monoclonal antibody (10 μg/animal twice a month, i.p.) or vehicle (10 μg/animal twice a month, i.p.). Panel **b**: immunofluorescence of hippocampi for CD3 (red) and FoxP3 (green) expression and co-localization from the same animal groups as above (merge column; DAPI = nuclear staining). The respective side columns are the lower magnification samples where the areas to analyze (CA1, CA2, CA3, and CA4) were magnified (framed in a green box). *WT* wild-type animals (*n* = 5/group).; AD: 3xTg-AD animals (*n* = 5/group)

To verify whether the expression of both GITR and FoxP3 in the hippocampus of 3xTg-AD mice was associated with lymphocytes infiltration, immunostaining for CD3 was performed in the CA2 and CA3 hippocampal areas of untreated 3xTg-AD mice revealing a substantial immunoreactivity in both areas as compared with wild type mice. On the other hand, CD3 immunoreactivity was reduced in the hippocampus of 3xTg-AD mice treated with the anti TNFSF10 antibody compared to untreated animals (Fig. 5a).

To better understand whether such decrease of CD3 immunoreactivity in the hippocampus of anti TNFSF10-treated animals could be due to a decreased number of Treg cell (as per the blunted expression of FoxP3 in the hippocampus), we performed specific immunofluorescence experiments and observed that FoxP3 expression decreased following anti TNFSF10 treatment, in parallel with decreased CD3 specific immunoreactivity (Fig. 5b). See Additional file 3: Figure S3 for negative controls.

### Expression of immune markers in the human AD brain

**Fig. 6:**
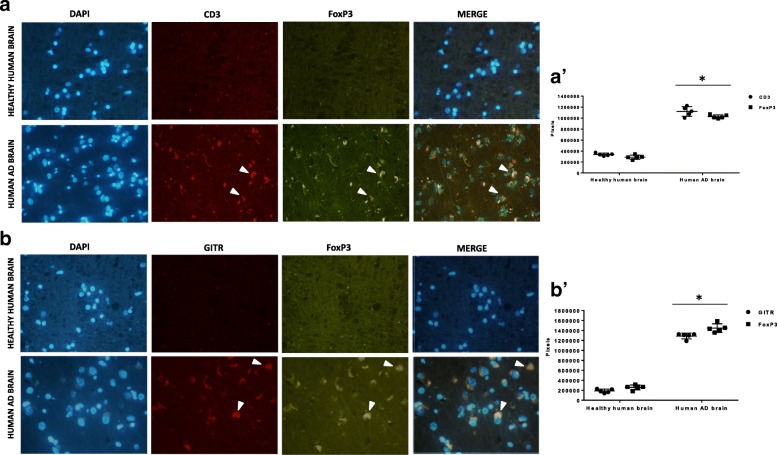
Confocal microscopy for detection of CD3, FoxP3, and GITR positive cells in the human AD brain (*n* = 4). Panel (a): CD3 (red) and FoxP3 (green) expression in the human AD brain and respective co-localization (white arrows; merge column; DAPI = nuclear staining). Panel (b): GITR (red) and Foxp3 (green) expression in the human AD brain and respective co-localization (white arrows; merge column; DAPI = nuclear staining). Panels (a’, b’): densitometric counts of immunopositive cells. *p < 0.001 vs. healthy human brain; (one-way ANOVA, followed by a Duncan’s multiple range test). Vertical bars are means ± S.E.M.

We asked whether changes in CD3, FoxP3, and GITR immunoreactivity observed in the hippocampus of the 3xTg-AD mice similarly occurred in the brain of AD patients. Interestingly, immunohistochemistry showed that while CD3 and FoxP3 were absent in in the hippocampus from healthy individuals (Fig. 6(a)), both proteins were detected in the hippocampus from AD brain, showing a co-localized immunostaining, consistently with the data obtained in mice (Fig. 6(a; white arrows)). Furthermore, the human AD hippocampus also expressed GITR, which co-localized with FoxP3 (Fig. 6(b; white arrows)). Respective densitometric counts are shown in panels a’ and b’. See Additional file 4: Figure S4 and Additional file 5: Figure S5 for negative controls.

### Reactive proinflammatory microglia is blunted by the anti-TNFSF10 treatment in 3xTg-AD mice

Chronically activated microglia and the related proinflammatory response play a pivotal role in AD neuropathology [10]. Here, we showed confocal images of the microglial marker CD11b, co-localized with the proinflammatory cytokine TNF-α in the hippocampus of mice treated as detailed above.

**Fig. 7:**
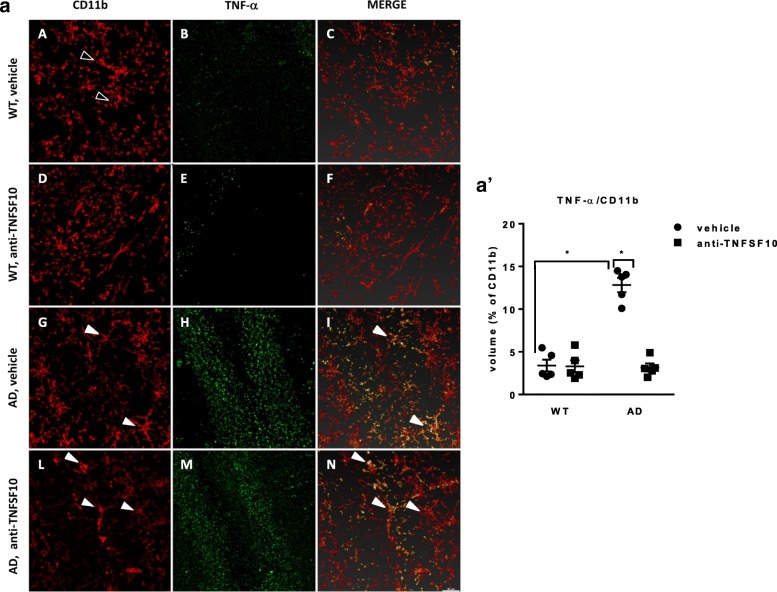
Confocal microscopy for detection of TNF-α and CD11b expression in the microglia of the CA2-CA3 regions of the hippocampus of 3xTg AD mice, following chronic treatment (12 months) with an anti-TNFSF10 monoclonal antibody (10 μg/animal twice a month, i.p.; (*n* = 10/group) or vehicle (10 μg/animal twice a month, i.p.). Representative images showing respectively CD11b (red, panels A, D, G, L), TNF-α (green, panels B, E, H, M), and TNF-α co-localized with CD11b (panels C, F, I, N). Side graphic represents the densitometric count of positive cells; *p ≤ 0.05 vs. all other groups; (two-way ANOVA, followed by Bonferroni post-hoc test). Vertical bars are means ± S.E.M. *WT* wild-type animals (*n* = 5/group); AD: 3xTg-AD animals (*n* = 5/group). Black arrows point resting microglia; white arrows point activated microglia (panels G and I), or co-localization TNF-α/CD11b (panels I and N)

Microglia in untreated 3xTg-AD mice showed an activated morphology, as suggested by the cell body enlargement and ticker processes while in wild-type mice microglia displayed a resting morphology. Moreover, untreated 3xTg-AD mice showed a dramatic increase in microglial TNF-α, i.e., TNFα co-localized with CD11b, as compared with WT mice (Fig. 7(a, A–C, G–I)). Of note, the treatment with anti-TNFSF10, significantly reduced TNF-α levels in microglia (Fig. 7(a, L–N)).

### Expression of Aβ and phosphorylated-tau protein (p-tau) are significantly attenuated in 3xTg-AD mice treated chronically with anti TNFSF10

**Fig. 8:**
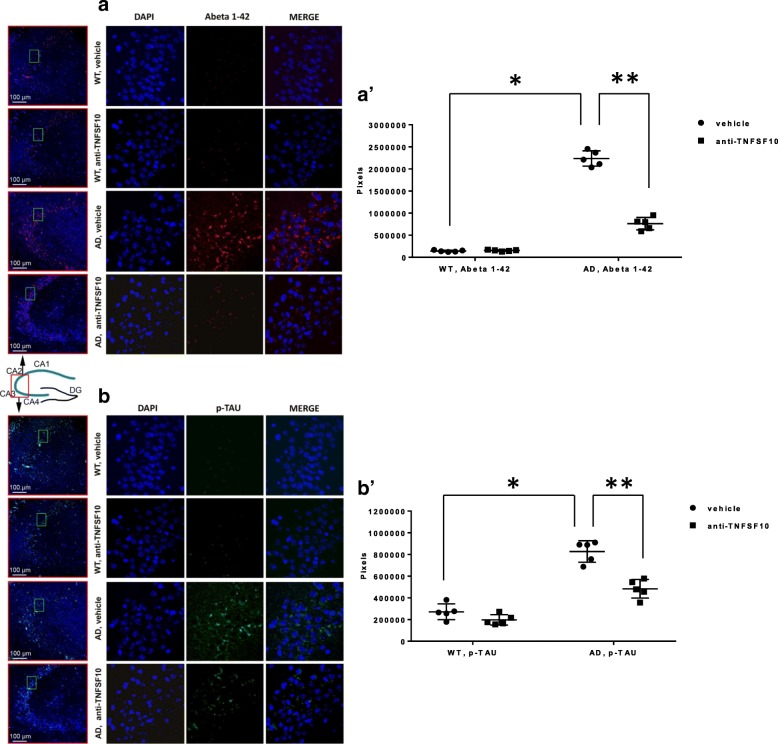
Effects of the treatment with an anti-TNFSF10 monoclonal antibody on the expression of either Aβ 1–42 (panel a) and phosphorylated Tau protein (p-TAU) (panel b) in the hippocampus of 3xTg-AD mice: representative immunofluorescence images of hippocampi from each animal group (merge column; DAPI = nuclear staining). The respective left-hand side columns are the samples where the areas to be magnified for analysis were picked (green frame boxes; CA1, CA2, CA3, and CA4). Panels (a’, b’): densitometric count of immune-positive cells, for, respectively, Aβ 1–42 and p-TAU. *p < 0.001 3xTg-AD mice untreated vs WT-vehicle; **p < 0.001 3xTg-AD mice treated with anti-TNFSF10 vs. untreated 3xTg-AD mice (one-way ANOVA, followed by a Duncan’s multiple range test). Vertical bars are means ± S.E.M. *WT* wild-type animals (*n* = 5/group); AD: 3xTg-AD animals (*n* = 5/group)

Amyloid beta and the phosphorylated tau protein are recognize hallmarks of AD, as it is well established that their amount correlates with the disease staging in the AD brain [30] as well as in the 3xTg-AD model [25]. In the light of our findings on the immune response in 3xTg-AD mice, we attempted to establish whether the expression of both Abeta 1–42 (Fig. 8(a)) and p-tau (Fig. 8(b)) paralleled the immune activity pattern observed in the spleen and in the hippocampus of 3xTg-AD mice. Immunohistochemical analysis showed that both amyloid beta 1–42 and p-tau were abundantly expressed in untreated 3xTg-AD mice, while their expression was greatly attenuated after treatment of the animals with the anti-TNFSF10 antibody (Fig. 8). See Additional file 6: Figure S6 and Additional file 7: Figure S7 for negative controls.

## Discussion

Here, we showed that 3xTg-AD mice with age-related cognitive decline manifest also an imbalance of the immune/inflammatory response.

We observed an increased spleen weight, as well as decreased brain and body weight in untreated 3xTg-AD mice in their advanced age, confirming data from other authors [29, 31]. At a first glance, one could argue that such spleen enlargement could be due to the presence of a transgene. However, the risk for genome insertion site-related phenotypes and consequent splenomegaly is highly unlikely, because APP_swe_ and PS1_M145V_ transgenes are expressed but not translated in the spleen. In fact, Oddo et al. [25] reported the absence of protein translates in various organs of the 3xTg-AD mouse, with the exception of the central nervous system. In addition, it has been reported that splenic alterations occur in 3xTg-AD mice fairly ahead of the onset of the amyloid pathology [29]. Accordingly, an altered rate of CD3 lymphocytes in the spleen has been reported, suggesting an autoimmune/inflammatory involvement [29]. In this line, light behavioral dysfunctions occurring in the early life of 3xTg-AD mice have been associated with the human parameters of minimal cognitive impairment (MCI), with episodic memory loss in the absence of major cognitive dysfunction [32].

Interestingly, an accumulation of human beta-amyloid 1–42 originating in the transgenic mouse brain was revealed in the spleens of 3xTg-AD mice by immunohistochemical and Western blot analysis. Although in this mouse model beta-amyloid 1–42 and tau transgenes are limited expressed by brain and spinal cord [25], different reports showed that beta-amyloid 1–42 and tau are also present in the blood and peripheral organs [33–36]. Therefore, we may speculate that over-accumulated brain Aβ might be transported to peripheral organs [37], thereby functioning as a signal to trigger peripheral immune responses [38]. In this scenario, beta-amyloid 1–42 deposits in the spleen could be responsible for the peripheral inflammation observed in the AD mice, and for changes in immune cell setting, with special regard to the increased CD3 population [31].

Consistently, we found that a set of inflammatory molecules, including TNFSF10, and its death receptor, as well as COX2, iNOS, IL-1β, and TNF-α were significantly increased in the spleen homogenates from 3xTg-AD mice. It is also noteworthy that the splenic expression of the antigen GITR [39], as well as that of the Treg cell-related transcription factor FoxP3 [40], were dramatically increased in the spleen of the same animals, whereas the expression of the anti-inflammatory cytokine IL-10 was negligible.

Now, keeping in mind the concept that molecules from the CNS, such as Aβ, may influence also peripheral immune/inflammatory response [41], the exceeding synthesis of Aβ in the brain could set into motion specific immune response, as suggested by the increased expression of splenic Treg markers GITR and FoxP3. In fact, it is noteworthy that Treg cells may suppress, for example, autoreactive T lymphocytes recruited in course of chronic inflammation [42], which could be envisioned as a sort of feedback mechanism to avoid overshooting of the immune/inflammatory response and tissue damage, as, for example, activation of glucocorticoid secretion set into motion by cytokines release in the course of an immune/inflammatory response [39].

Although contrasting data are available on the role of the GITR system in Treg setting [43], nevertheless, more recent research has defined its role as a marker for human Treg cells [44], confirmed by data showing that GITR could be regarded as a receptor belonging to the immune checkpoint family [43]. In fact, it is known that when Treg cell function is pharmacologically inhibited by the immune checkpoint inhibitors, the restoration of a balanced immune response is achieved, due to inactivation of the Treg cells enabled by tumors [45].

TNFSF10 produced by macrophages is known to intervene in immune processes, such as graft-versus-host disease (GVHD), protection of privileged barriers, and others [16]. In the same line, our results indicated that high splenic expression of TNFSF10 was associated with an increased GITR and FoxP3 expression in aged 3xTg-AD mice. Consistently, the treatment with the anti-TNFSF10 antibody resulted in a decreased expression of Treg cells markers and in a significant attenuation of inflammatory molecules COX2, iNOS, IL-1β, and TNF-α, with shrunk splenic volume. Similarly, there is evidence that PD-1 checkpoint inhibitors can reduce brain pathology and improve cognition in the AD murine model [46].

Our data indeed confirm the potent inflammatory properties of TNFSF10, mediated by its death receptor TNFRSF10B [19], in peripheral organs, in which a chronic inflammatory/immune response was set into motion by a noxious challenge, such as Aβ, and support the value of TNFSF10 neutralization in shutting the inflammatory process down [19, 47, 48].

Evidence shows that peripheral lymphocytes, including Treg cells, are able to crawl across the BBB in neuropathologic conditions [49]. In this line, we have previously shown that TNFSF10 is substantially expressed in both the human AD brain [50], as well as in the hippocampus of 3xTg-AD mice [19], and that its immunoneutralization by means of a monoclonal antibody against TNFS10, is associated with an almost complete recovery of cognitive capacities, along with blunted expression of inflammatory mediators in the brain [19].

Although poor evidence is currently available on the influence of peripheral immune response upon the AD brain pathology [23, 24], recent data indicate that not only peripheral immunocytes have privileged gates to enter the brain [51] but also they can significantly influence the progression of brain pathology in murine models of AD [12]. More recently, a relationship has been demonstrated between adaptive immune-related impairment and AD neuropathology in the 3xTg-AD mouse model, suggesting a causal role for typical Aβ and tau pathologies [38].

Here, we showed high expression of FoxP3 and GITR and their co-localization in the hippocampus of untreated 3xTg-AD mice while their expression was attenuated by treatment with the anti-TNFSF10 antibody, suggesting that neutralization of TNFSF10, known to increase the number and activity of Treg cells [16], achieves a significant anti-inflammatory effect. In light of the capability of TNFSF10 of recruiting other immune/inflammatory mediators during the neurodegenerative process [19, 47], it is noteworthy that also proinflammatory mediators COX2, iNOS, IL-1β, and TNF-α were downregulated by anti-TNFSF10 treatment along with the increased levels of the anti-inflammatory cytokine IL-10, [52], which is also produced by activated microglia [53], injured neurons [54], and other cell types in the CNS. In fact, it appears that IL-10 works as a protective tool in course of brain damage, likely to avoid propagation of neuroinflammatory areas and related detrimental effects [55].

We also found that CD3 positive cells were significantly represented in the hippocampus of untreated 3xTg-AD animals, eventually returning to their basal expression after the anti-TNFSF10 treatment. In addition, we also demonstrated that FoxP3-specific immunofluorescence was consistent with that of CD3. As postulated by other authors, FoxP3-positive cells crossing into the brain could create favorable conditions for an overshooting immune/inflammatory response to Aβ [12]. In a similar line, a role for Treg cells has been identified in other tissues chronically inflamed and in models of impaired immune response [16, 56, 57]. This may partly account for the significant attenuation of the inflammatory molecules in the brain obtained by neutralization of TNFSF10, which also recruits Treg cells [16], Interestingly, we showed that the human post-mortem AD brain expressed CD3 (which was not expressed by the healthy human brain), that co-localized with FoxP3 and GITR, corroborating the hypothesis that the AD brain is also characterized by the presence of immunocytes, in analogy with other immune-related CNS disorders [49].

Although there are no clinical data linking AD to splenomegaly, prominent inflammatory and innate immune responses have been observed in both AD and minimal cognitive impairment (MCI) [58–60]. Accordingly, there are reports that suggest that circulating plasma levels of cytokines are increased in AD [61, 62]. Moreover, increased plasma levels of TNF-α have been associated with scores in MMS [62]. More recently, an increase in the CD4/CD8 ratio in 3xTg-AD mice was reported [38], suggesting a deficit in the adaptive immune response consistently with data reporting aberrant lymphocyte populations in AD individuals [63–68] .

Comparing the immunological scenario in the 3xTg-AD animals with the data from human brains, it might be speculated that neutralization of TNFSF10 may produces beneficial effects through two possible mechanisms. The first one relates to the decreased expression of the inflammatory/immune mediators [16], with a second alternative mechanism relating to the removal of the Treg influence on immunocytes, allowing a re-balance of the immune response and reduced brain accumulation of Aβ [12].

The hypothesis of the restoration of a proper immune reactivity in the hippocampus of 3xTg-AD mice by the anti-TNFSF10 treatment was corroborated by the demonstration that proinflammatory microglia in 3xTg-AD mice was blunted after anti-TNFSF10 treatment in the same area. As known, activated microglia produces, in addition to TNFSF10 [69], several inflammatory cytokines included TNF-α [28], contributing to accelerate neuronal death [70]. Interestingly, microglia still displayed an activated morphology after anti-TNFSF10, which likely relates to an increased production of the anti-inflammatory cytokine IL-10 by these cells [27] . There is extensive evidence that microglia is activated by noxious stimuli within the CNS [71], including Aβ [72]. In addition, activated microglia has been show to influence both humoral and cell-mediated peripheral immune response [73], through the release of cytokines including TNF-α that may cross the BBB in the course of neurodegenerative processes [49]. This clearly support the hypothesis that soluble inflammatory molecules may influence tissues distant from the site of production [74]. Overall, this result suggests that the anti-TNFSF10 treatment induced a dampening of proinflammatory detrimental microglia, while boosting the production of anti-inflammatory molecules.

Restraint of the overall inflammatory/immune response achieved by the anti-TNFSF10 treatment came along with significantly decreased levels of both Aβ and p-Tau protein. Immunofluorescence data showed significant decrease of Aβ amounts in the hippocampus of 3xTg-AD mice treated with the anti-TNFSF10 antibody, in parallel with significant decrease of another AD hallmark, the hyperphosphorylated Tau protein, known to substantially contribute to neuronal death [61]. Cognitive decline is associated with a progressively increasing amount of both proteins in the hippocampus of 3xTg-AD animals [19]. Assuming that the Aβ excess induces TNFSF10 expression in neurons [13], we suggest that neutralization of TNFSF10 represents a potential strategy to limit Aβ production, with consistent subsequent improvement of the cognitive outcome in AD [19].

## Conclusions

In conclusion, the neurotoxic effects of TNFSF10 may partly ensue from an unbalanced equilibrium of the overall immune response, triggered by Aβ which accumulates in both the CNS and in the spleen. Aβ may be, at any rate, the candidate antigenic challenge setting into motion a systemic immune response associated with neuroinflammation. Re-trafficking of immunocytes between the periphery and the brain [46] of 3xTg-AD mice might contribute to neuroinflammation, with the involvement of Treg cells. Neutralization of TNFSF10 may significantly attenuate its detrimental effects along with those of other inflammatory mediators in the brain; on the other hand, decreased Treg cells in both the periphery and the brain may enhance the activity of other cells such as macrophages/microglia and monocytes [12], to efficiently clear Aβ from the brain tissue [75].

Finally, our data demonstrate that TNFSF10 substantially cooperates with other cytokines in sustaining inflammation in the 3xTg-AD mouse brain. TNFSF10 also inhibits the beneficial activity of the immune response by recruiting Treg cells, resulting in the inhibition of the anti-inflammatory machinery. Blockade of TNFSF10 may thus be envisioned as an innovative treatment of neurodegeneration in AD.

## Additional files

Additional file 1: **Figure S1.** Effects of the treatment with an anti-TNFSF10 monoclonal antibody on the expression of GITR and FoxP3, as well as the anti-inflammatory protein IL-10 in the hippocampus of 3xTg-AD mice. Representative immunofluorescence images of hippocampi for GITR and FoxP3 expression and co-localization from the same animal groups as above (merge column; DAPI = nuclear staining). Negative controls are reported in all panels marked with acronyms of secondary antibodies labeled with, respectively, Texas Red (TR) and Fluorescineisothiocyanate (FITC). (PDF 526 kb)

Additional file 2: **Figure S2.** Effects of the treatment with an anti-TNFSF10 monoclonal antibody on the expression of FoxP3, as well as the anti-inflammatory protein IL-10 in the hippocampus of 3xTg-AD mice. Immunofluorescence by confocal microscopy of hippocampi for IL-10 and FoxP3 expression and co-localization from the same animal groups as above (merge column; DAPI = nuclear staining). WT: wild type animals; AD: 3xTg-AD animals; veh: vehicle. Negative controls are reported in all panels marked with acronyms of secondary antibodies labeled with, respectively, Texas Red (TR) and Fluorescineisothiocyanate (FITC). (PDF 428 kb)

Additional file 3: **Figure S3.** Confocal microscopy for detection of CD3 positive cells in the hippocampus of 3xTg AD mice, following chronic treatment (12 months) with an anti-TNFSF10 monoclonal antibody (10 μg/animal twice a month, i.p.). Representative immunofluorescence sections of hippocampi for CD3 and FoxP3 expression and co-localization from the same animal groups as above (merge column; DAPI = nuclear staining). WT: wild type animals; AD: 3xTg-AD animals; anti-TNFSF10: monoclonal anti-TNFSF10 antibody. Negative controls are reported in all panels marked with acronyms of secondary antibodies labeled with, respectively, Texas Red (TR) and Fluorescine isothiocyanate (FITC). (PDF 672 kb)

Additional file 4: **Figure S4.** Co-localization of GITR and Foxp3 in the human AD brain. Immunofluorescence in representative samples for both molecules was detected in immune cells in the hippocampus of AD patients, whereas it was practically absent in the brain of healthy individuals (merge column; DAPI = nuclear staining). Negative controls are reported in all panels marked with acronyms of secondary antibodies labeled with, respectively, Texas Red (TR) and Fluorescein isothiocyanate (FITC). (PDF 693 kb)

Additional file 5: **Figure S5.** Co-localization of CD3 and FoxP3 in the human AD brain. Immunofluorescence in representative samples for both molecules was detected in immune cells in the hippocampus of AD patients, whereas it was absent in the brain of healthy individuals (merge column; DAPI = nuclear staining). Negative controls are reported in all panels marked with acronyms of secondary antibodies labeled with, respectively, Texas Red (TR) and Fluorescein isothiocyanate (FITC). (PDF 842 kb)

Additional file 6: **Figure S6.** Negative controls for Fig. 8, panel a (Aβ1–42 expression). Negative controls are reported in all panels marked with acronyms of secondary antibodies labeled with, respectively, Texas Red (TR) (PDF 455 kb)

Additional file 7: **Figure S7.** Negative controls for Fig. 8, panel b (phosphorylated Tau protein expression). Negative controls are reported in all panels marked with acronyms of secondary antibodies labeled with, Alexa Fluor 488. (PDF 491 kb)

Additional file 8: **Table S1.** List of all antibodies used, with respective working dilutions for either WB or IHF, as well as Companies of origin and catalog number specification. (PDF 290 kb)

## Data Availability

The dataset used and analyzed during the current study are included within the article and its additional files. All material used in this manuscript will be made available to researcher subject to confidentiality.

## Abbreviations

AD: Alzheimer’s disease
APPswe: Swedish mutation of the amyloid precursor protein
Aβ: Amyloid beta
BBB: Blood-brain barrier
CD11b: Cluster of differentiation 11b
CD3: Cluster of differentiation 3
CNS: Central nervous system
COX2: Cyclooxygenase-2
FoxP3: Forkhead box P3
GITR: Glucocorticoid-induced
i.p: Intraperitoneally
IL-10: Interleukin-10;
IL-1β: Interleukin-1 beta
iNOS: Inducible nitric oxide synthase
PD-1: Programmed cell death protein 1
Psen1: Presenilin-1
p-Tau: Phosphorylated-tau protein
TNF: Receptor-related protein
TNFSF10: Tumor necrosis factor (ligand) superfamily, member 10
TNF-α: Tumor necrosis factor-alpha
Treg: Regulatory T cells
WT: Wild-type
