# Supplementary figures and images for: Beneficial effects of curtailing immune susceptibility in an Alzheimer’s disease model
Source: J Neuroinflammation. 2019 Aug 13;16:166. doi: 10.1186/s12974-019-1554-9 (PMC6693231; doi:10.1186/s12974-019-1554-9)

WT, vehicle

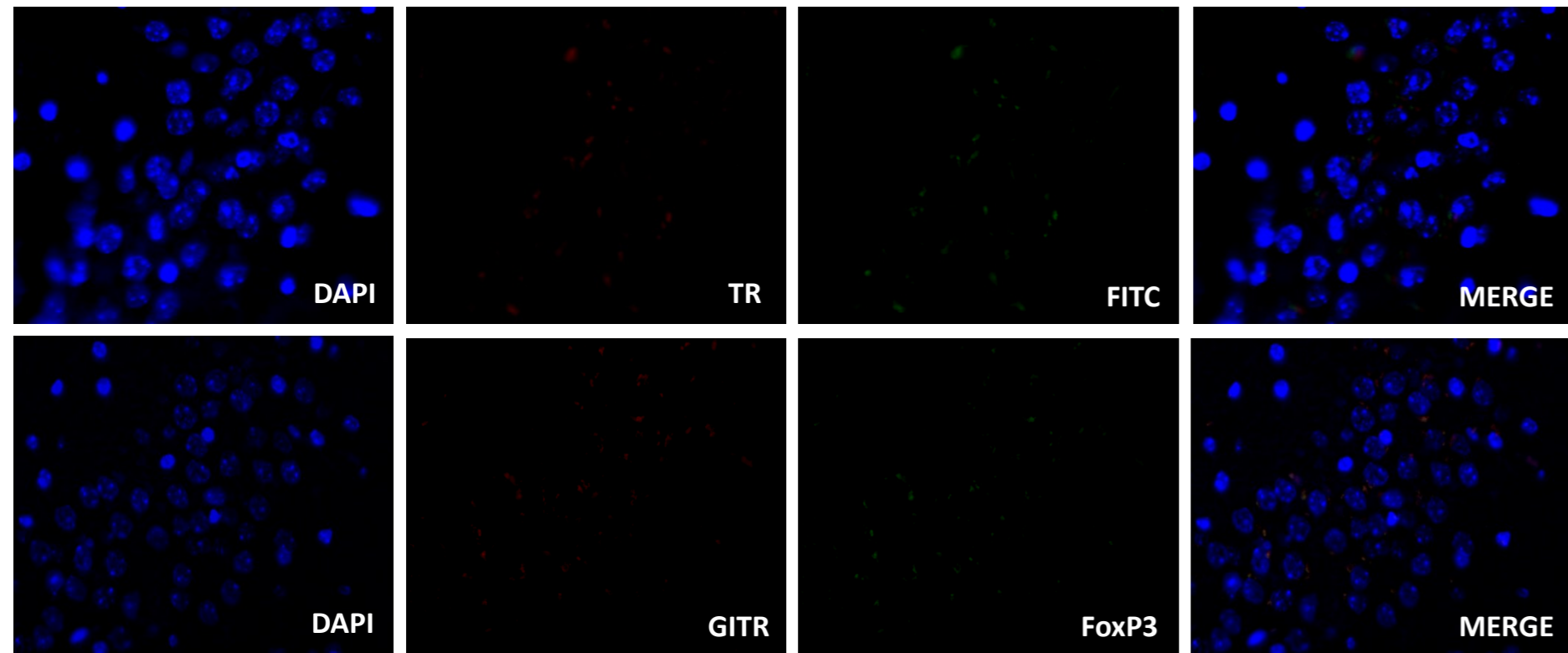

WT, anti-TNFSF10

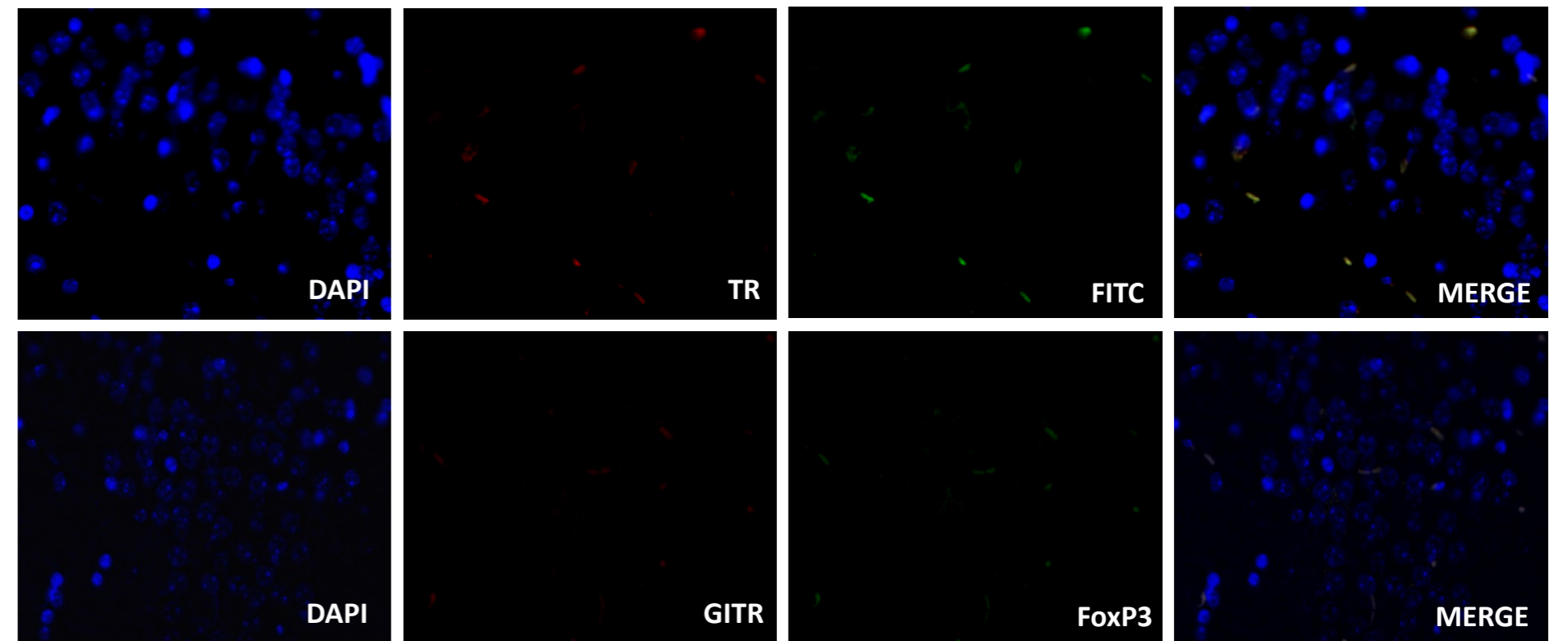

AD, vehicle

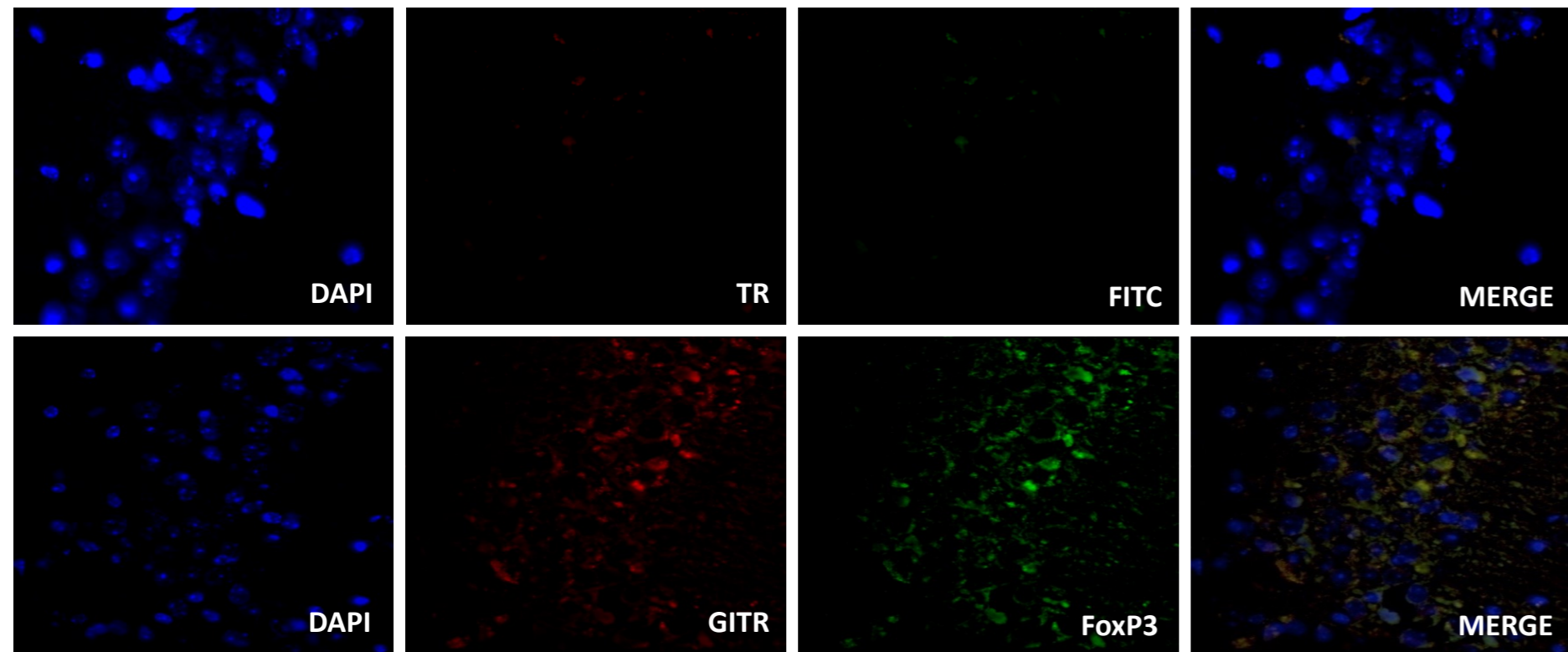

AD, anti-TNFSF10

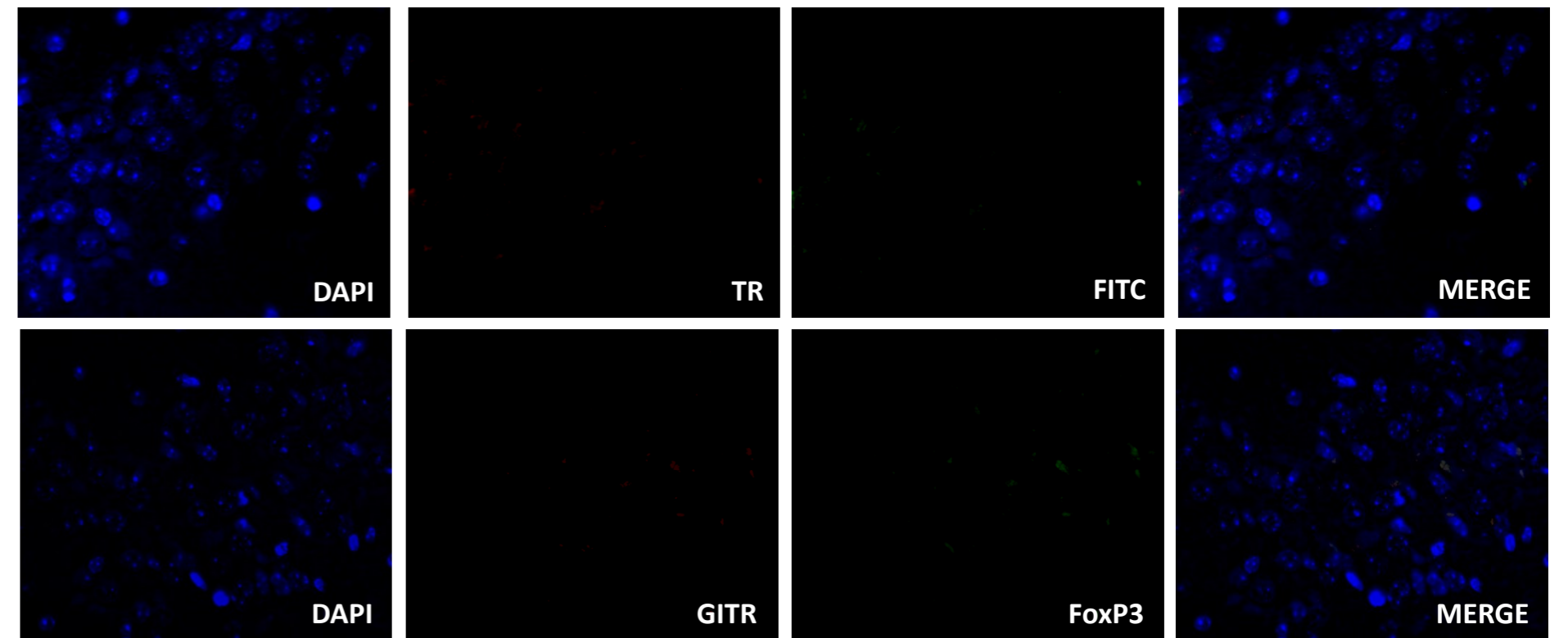

Supplement: Supplementary file 1 — Figure S1. Effects of the treatment with an anti-TNFSF10 monoclonal antibody on the expression of GITR and FoxP3, as well as the anti-inflammatory protein IL-10 in the hippocampus of 3xTg-AD mice. Representative immunofluorescence images of hippocampi for GITR and FoxP3 expression and co-localization from the same animal groups as above (merge column; DAPI = nuclear staining). Negative controls are reported in all panels marked with acronyms of secondary antibodies labeled with, respectively, Texas Red (TR) and Fluorescineisothiocyanate (FITC). (PDF 526 kb) [file 12974_2019_1554_MOESM1_ESM.pdf]

WT, vehicle

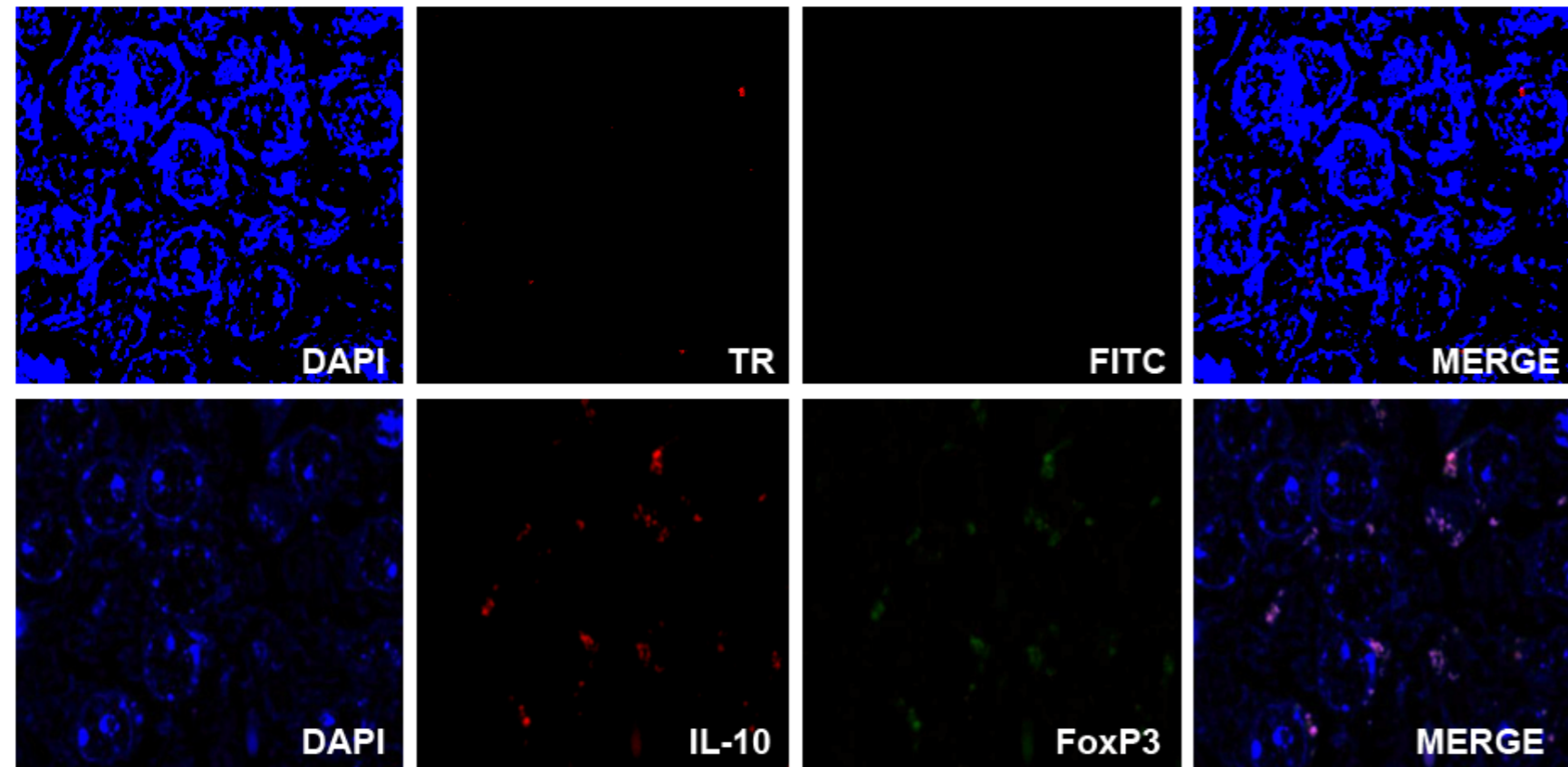

WT, anti-TNFSF10

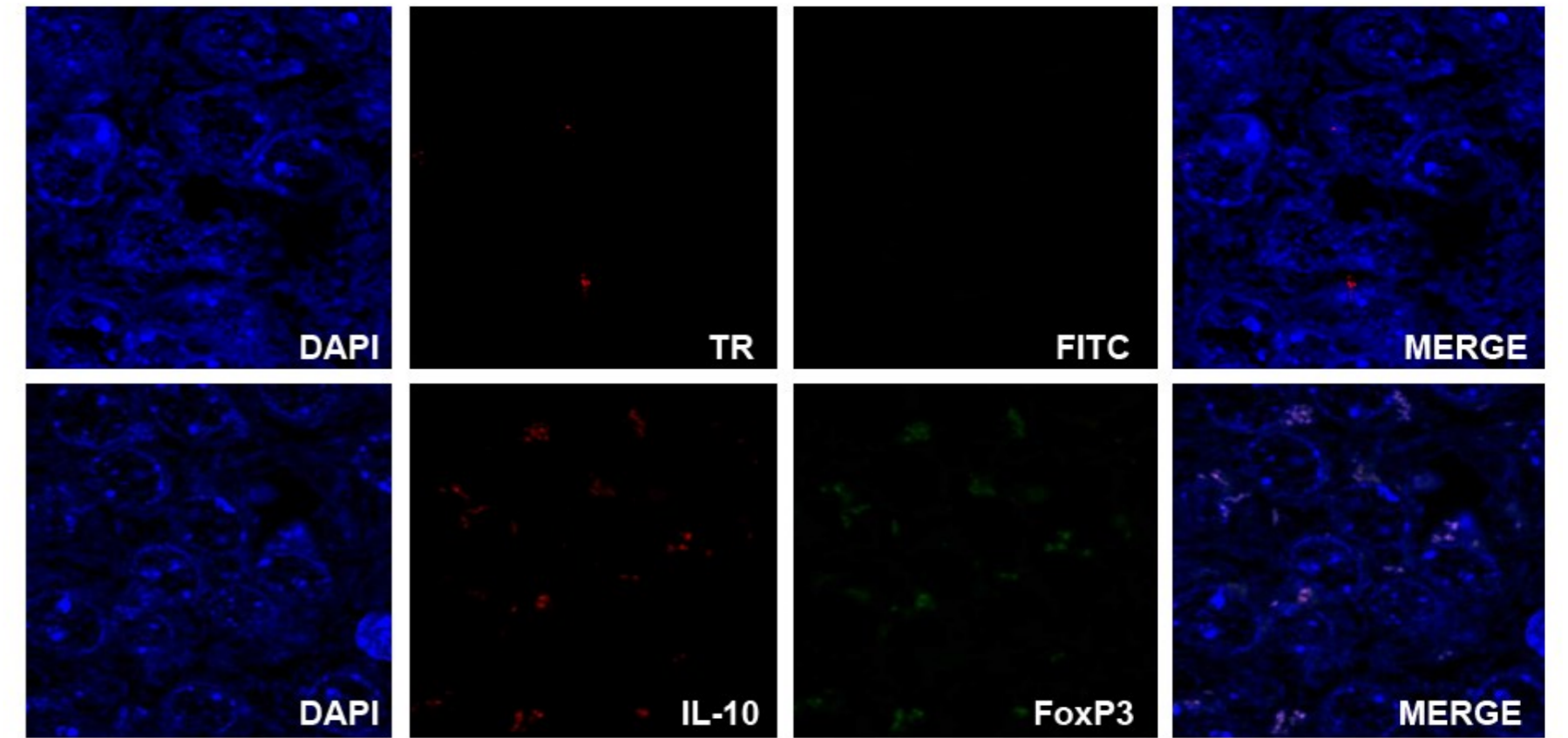

AD, vehicle

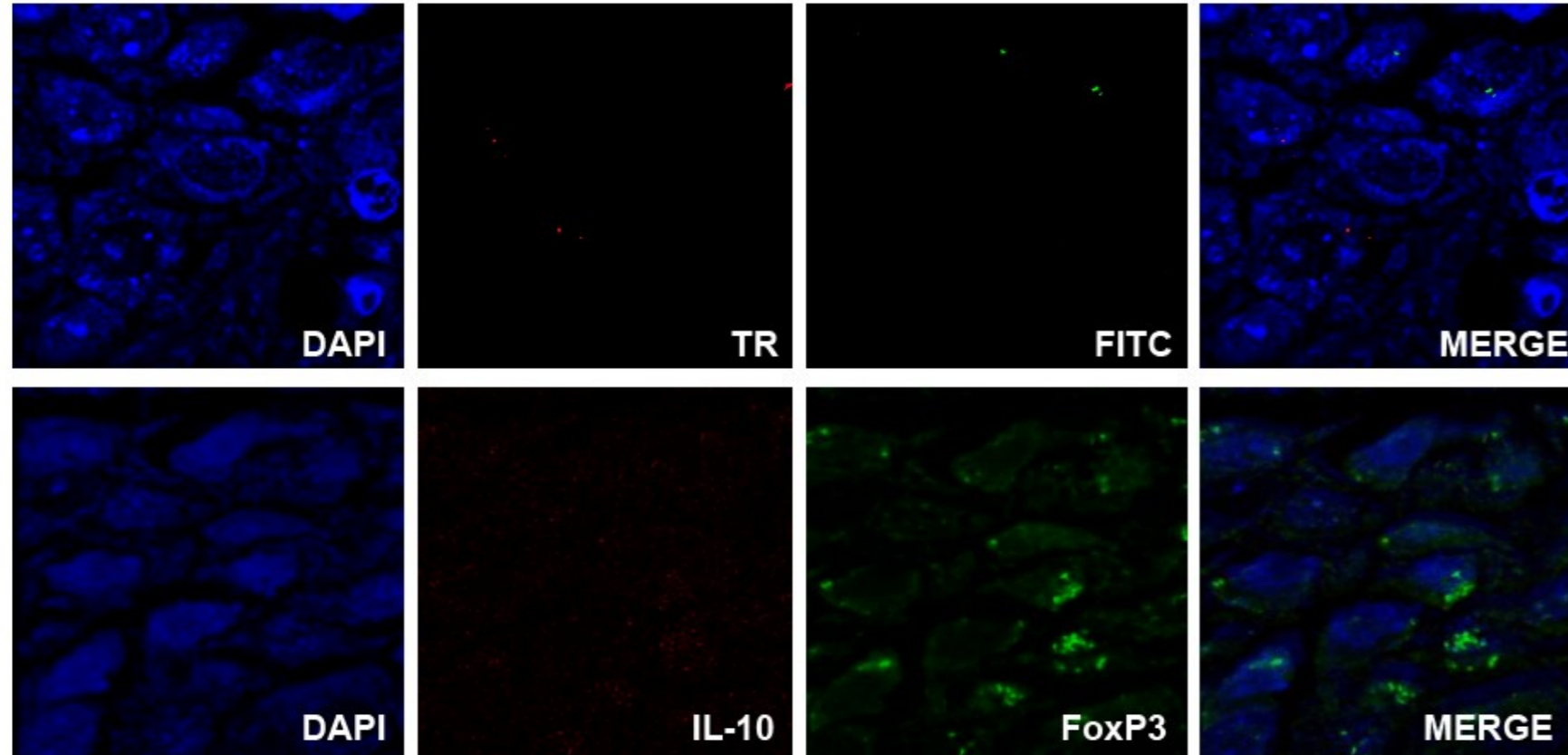

AD, anti-TNFSF10

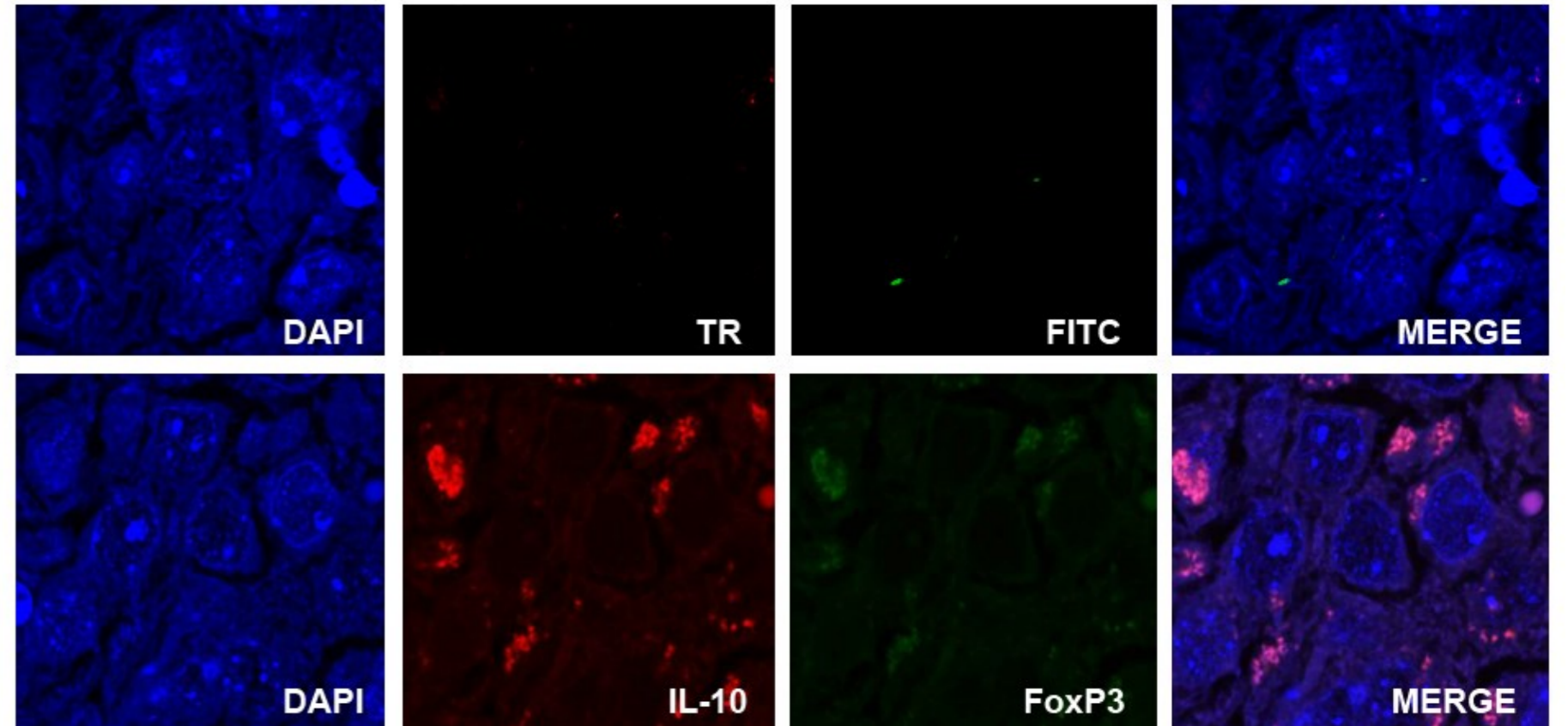

Supplement: Supplementary file 2 — Figure S2. Effects of the treatment with an anti-TNFSF10 monoclonal antibody on the expression of FoxP3, as well as the anti-inflammatory protein IL-10 in the hippocampus of 3xTg-AD mice. Immunofluorescence by confocal microscopy of hippocampi for IL-10 and FoxP3 expression and co-localization from the same animal groups as above (merge column; DAPI = nuclear staining). WT: wild type animals; AD: 3xTg-AD animals; veh: vehicle. Negative controls are reported in all panels marked with acronyms of secondary antibodies labeled with, respectively, Texas Red (TR) and Fluorescineisothiocyanate (FITC). (PDF 428 kb) [file 12974_2019_1554_MOESM2_ESM.pdf]

WT, vehicle

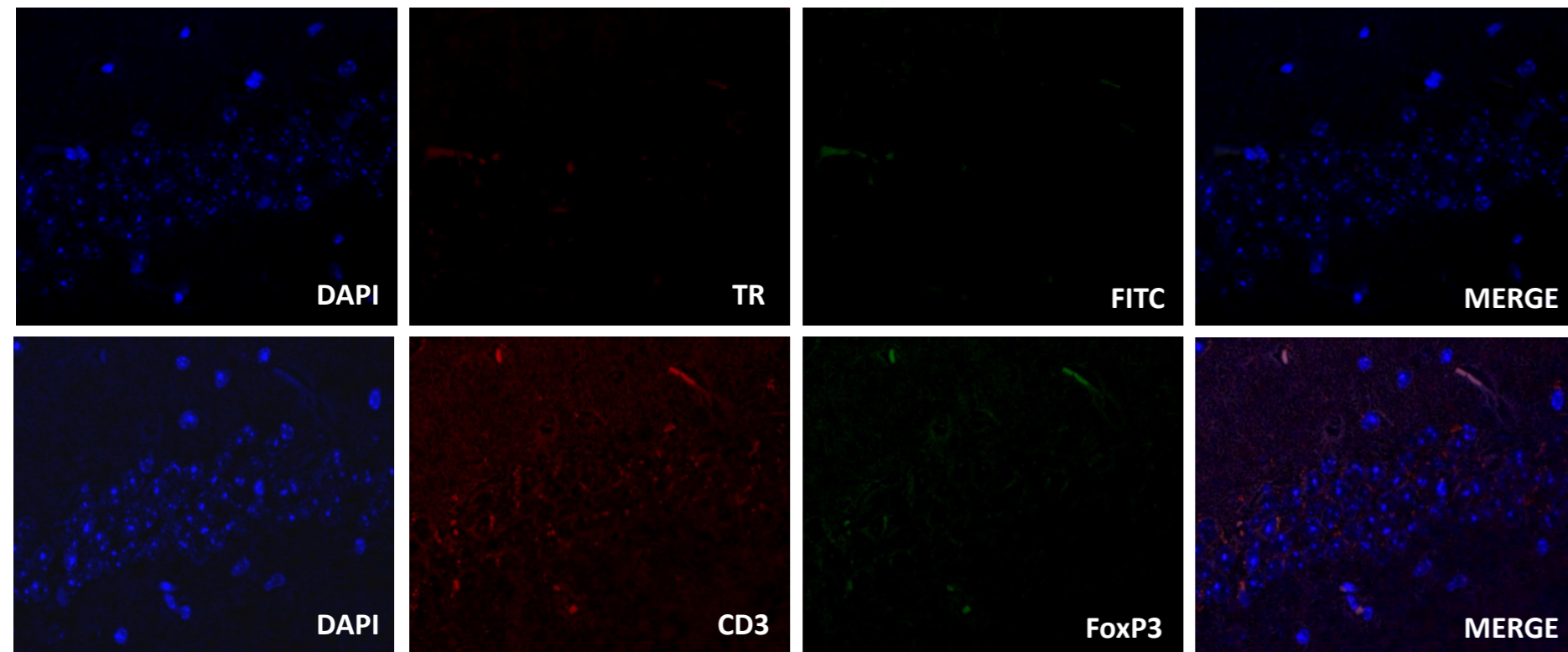

WT, anti-TNFSF10

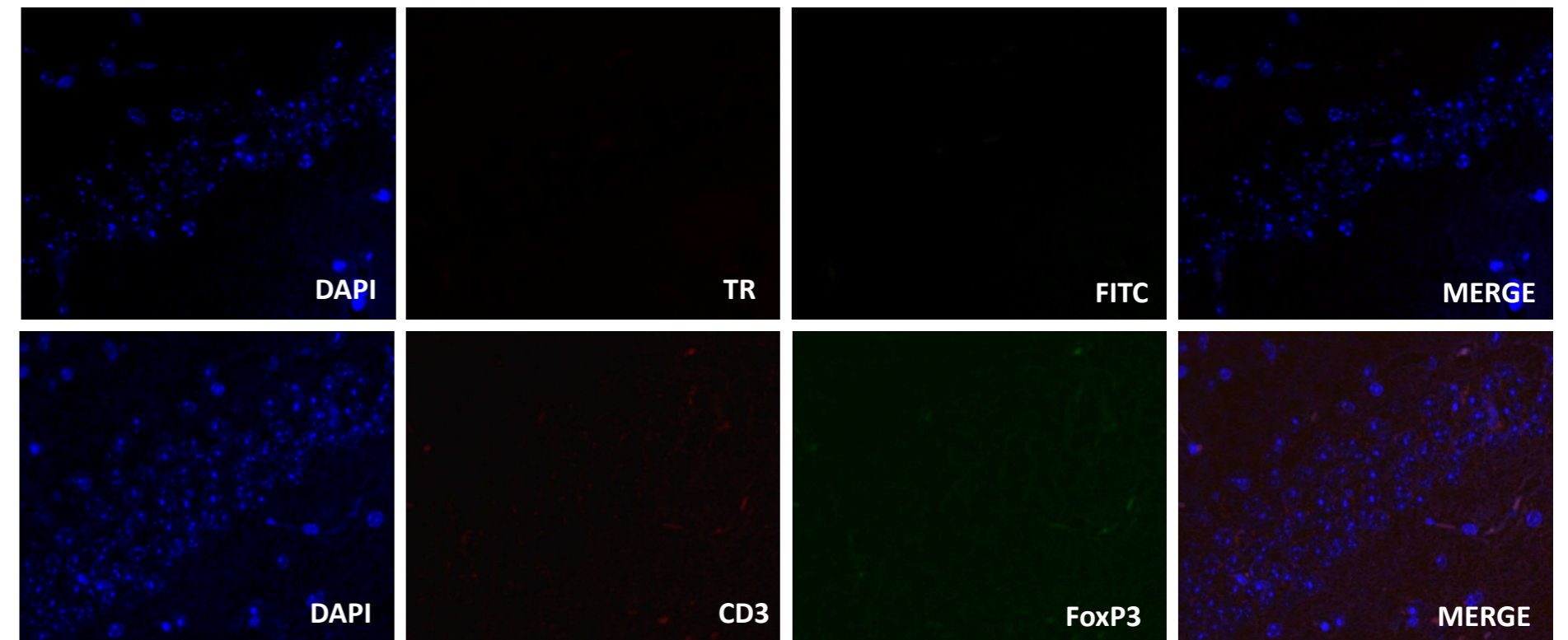

AD, vehicle

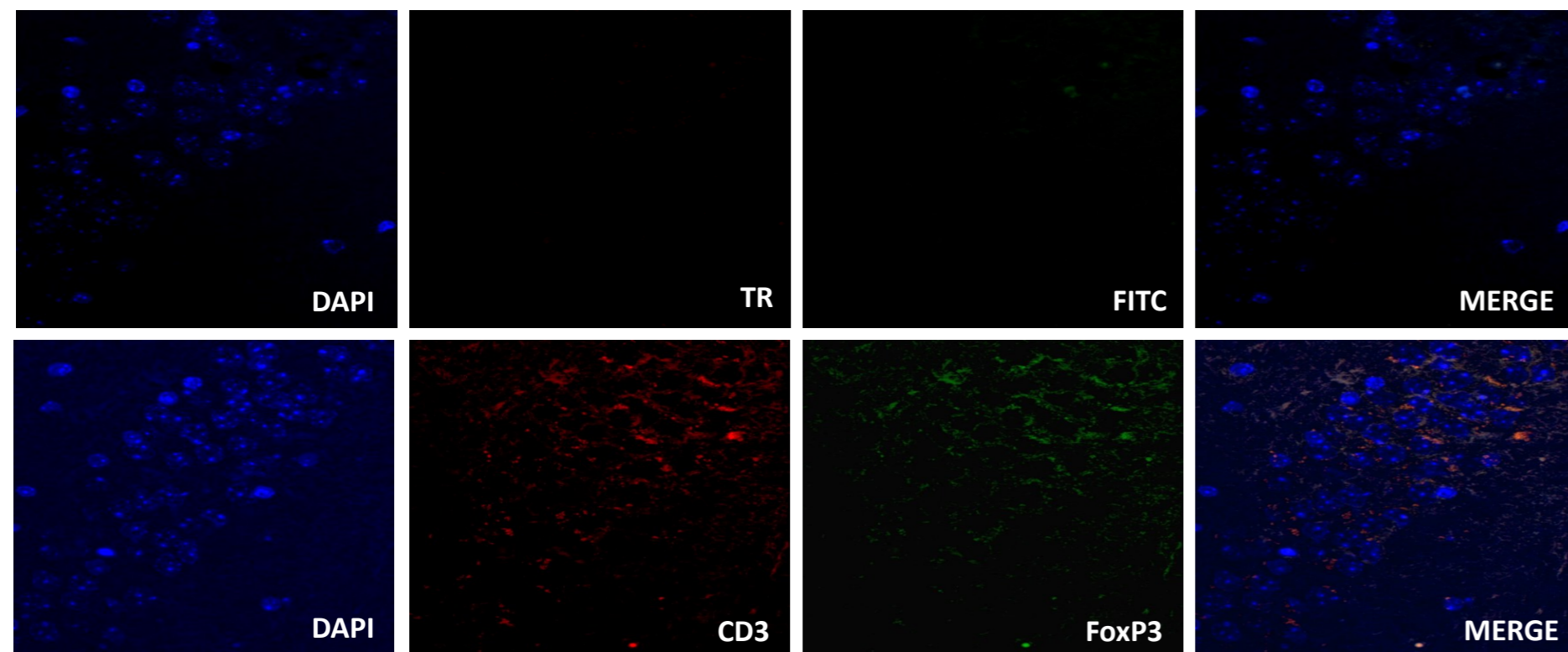

AD, anti-TNFSF10

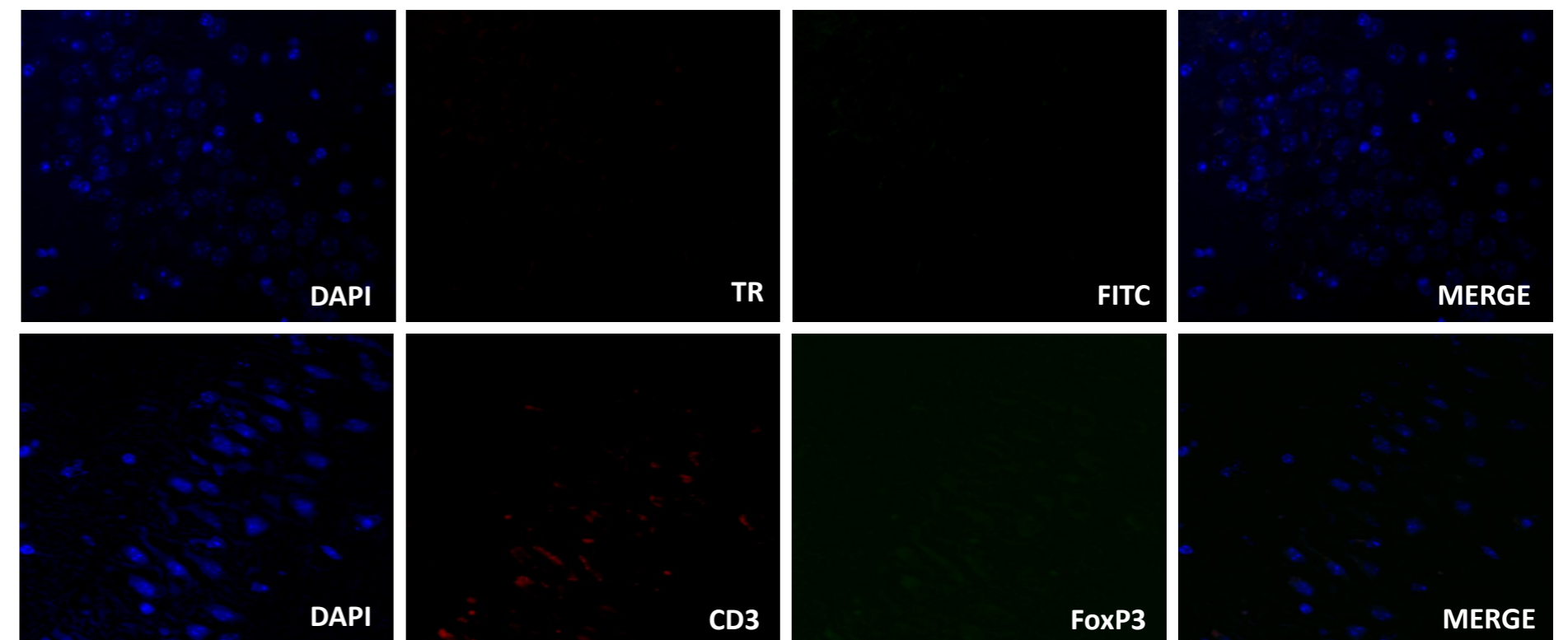

Supplement: Supplementary file 3 — Figure S3. Confocal microscopy for detection of CD3 positive cells in the hippocampus of 3xTg AD mice, following chronic treatment (12 months) with an anti-TNFSF10 monoclonal antibody (10 μg/animal twice a month, i.p.). Representative immunofluorescence sections of hippocampi for CD3 and FoxP3 expression and co-localization from the same animal groups as above (merge column; DAPI = nuclear staining). WT: wild type animals; AD: 3xTg-AD animals; anti-TNFSF10: monoclonal anti-TNFSF10 antibody. Negative controls are reported in all panels marked with acronyms of secondary antibodies labeled with, respectively, Texas Red (TR) and Fluorescine isothiocyanate (FITC). (PDF 672 kb) [file 12974_2019_1554_MOESM3_ESM.pdf]

**HEALTHY HUMAN BRAIN**

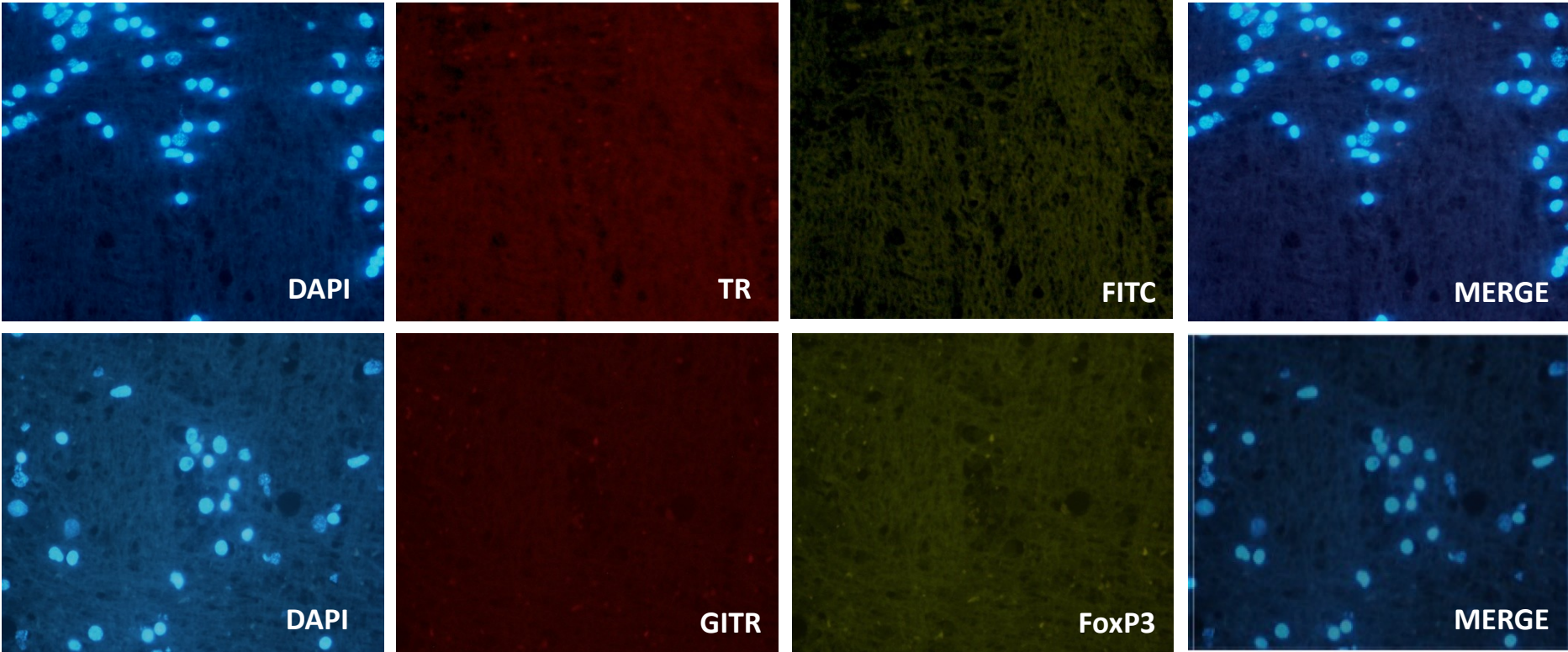

**HUMAN AD BRAIN**

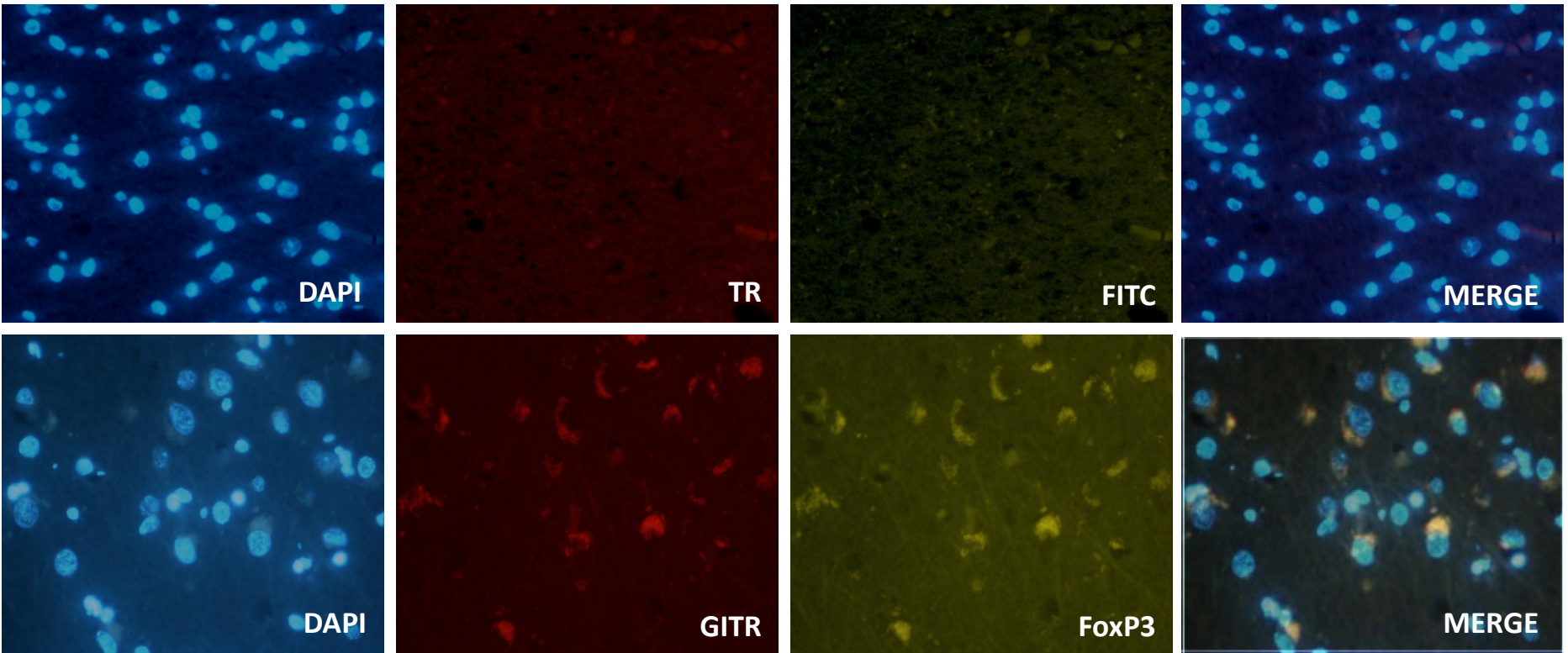

Supplement: Supplementary file 4 — Figure S4. Co-localization of GITR and Foxp3 in the human AD brain. Immunofluorescence in representative samples for both molecules was detected in immune cells in the hippocampus of AD patients, whereas it was practically absent in the brain of healthy individuals (merge column; DAPI = nuclear staining). Negative controls are reported in all panels marked with acronyms of secondary antibodies labeled with, respectively, Texas Red (TR) and Fluorescein isothiocyanate (FITC). (PDF 693 kb) [file 12974_2019_1554_MOESM4_ESM.pdf]

## HEALTHY HUMAN BRAIN

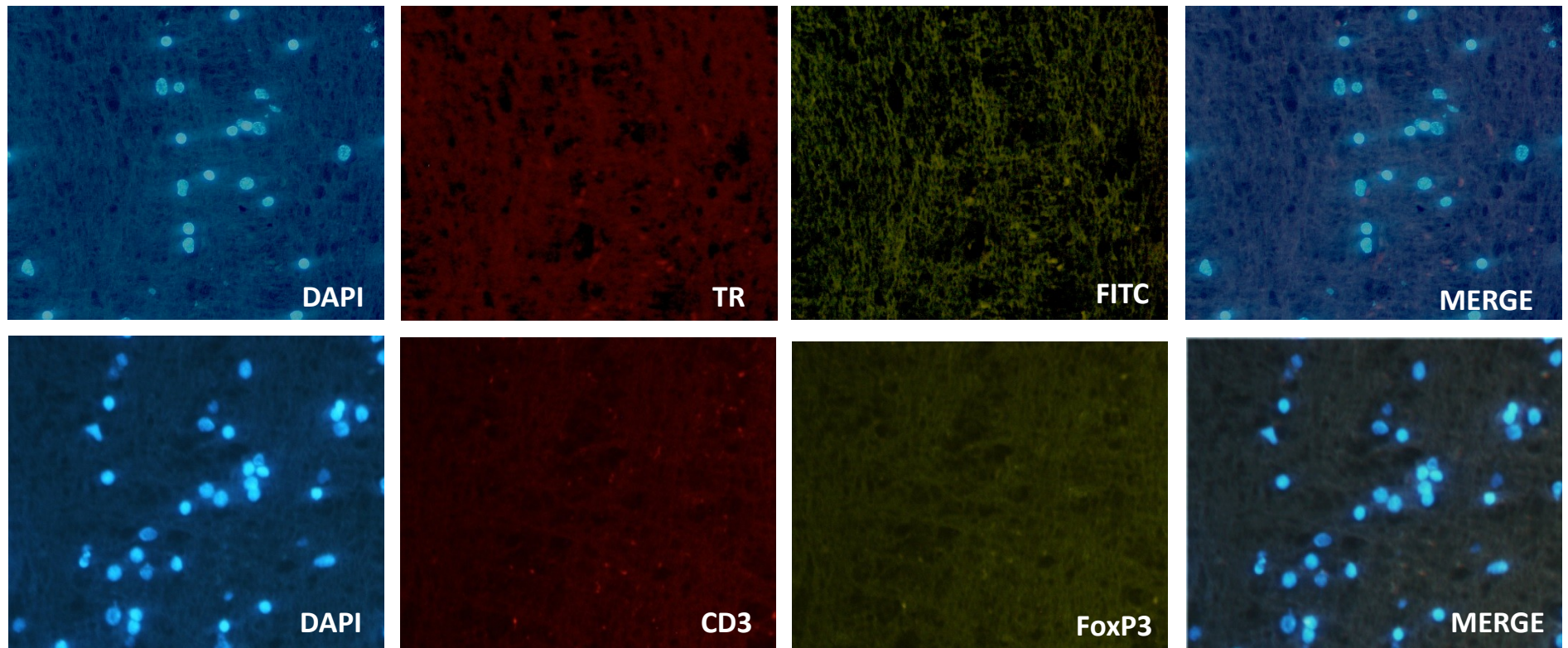

## HUMAN AD BRAIN

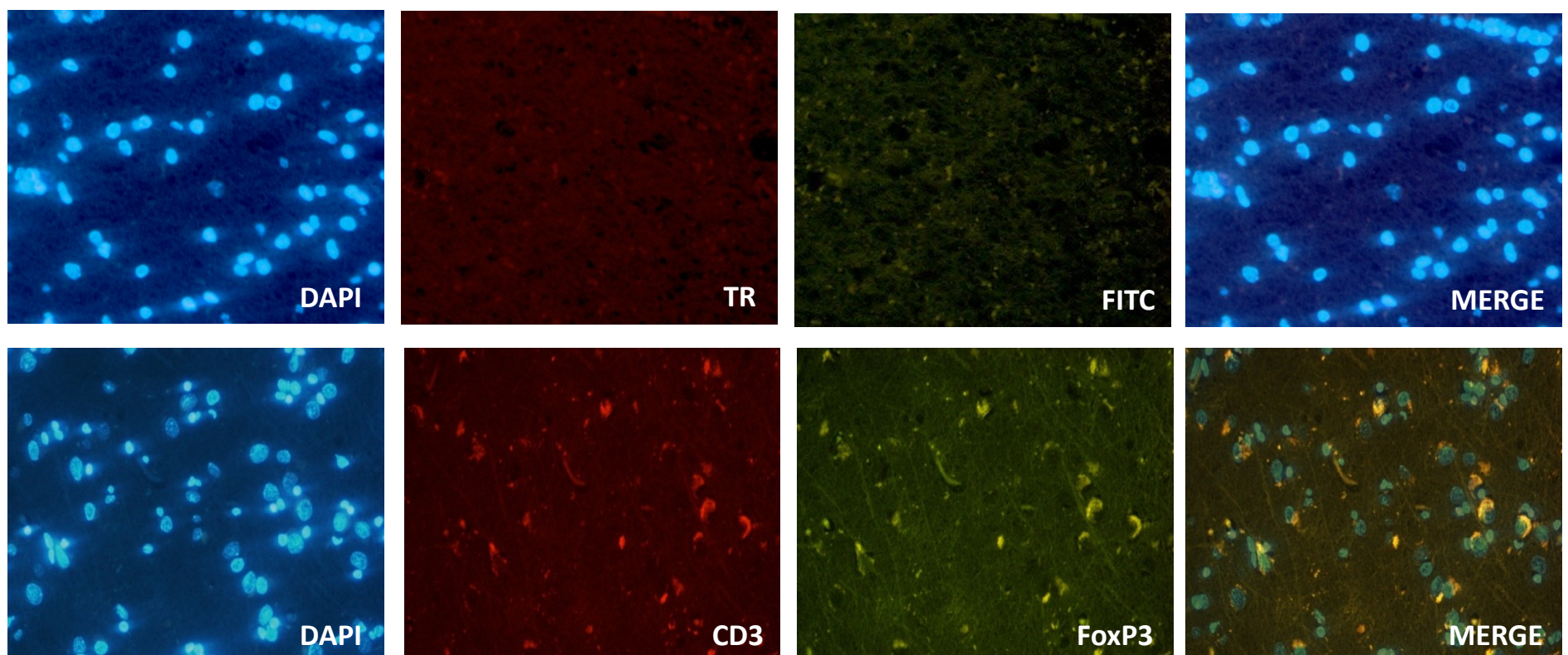

Supplement: Supplementary file 5 — Figure S5. Co-localization of CD3 and FoxP3 in the human AD brain. Immunofluorescence in representative samples for both molecules was detected in immune cells in the hippocampus of AD patients, whereas it was absent in the brain of healthy individuals (merge column; DAPI = nuclear staining). Negative controls are reported in all panels marked with acronyms of secondary antibodies labeled with, respectively, Texas Red (TR) and Fluorescein isothiocyanate (FITC). (PDF 842 kb) [file 12974_2019_1554_MOESM5_ESM.pdf]

WT, vehicle

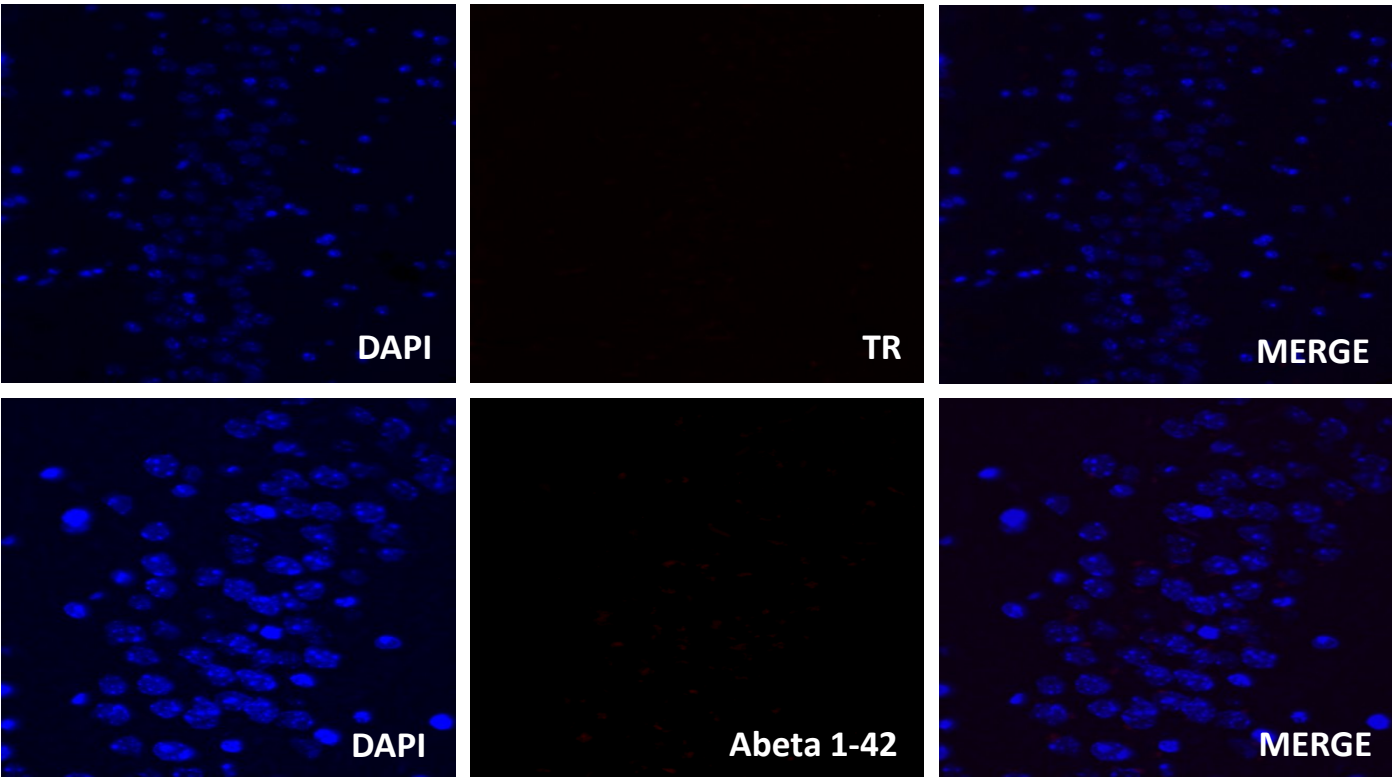

WT, anti-TNFSF10

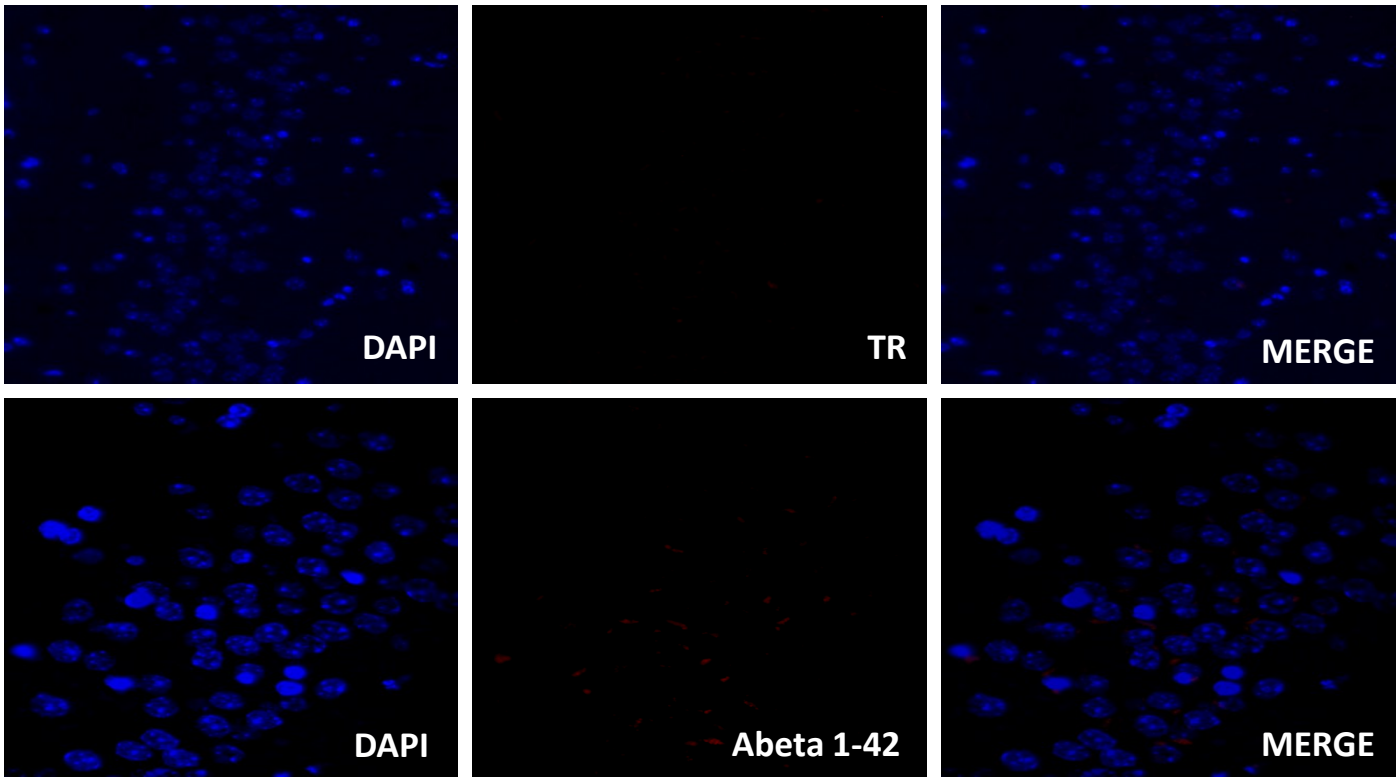

AD, vehicle

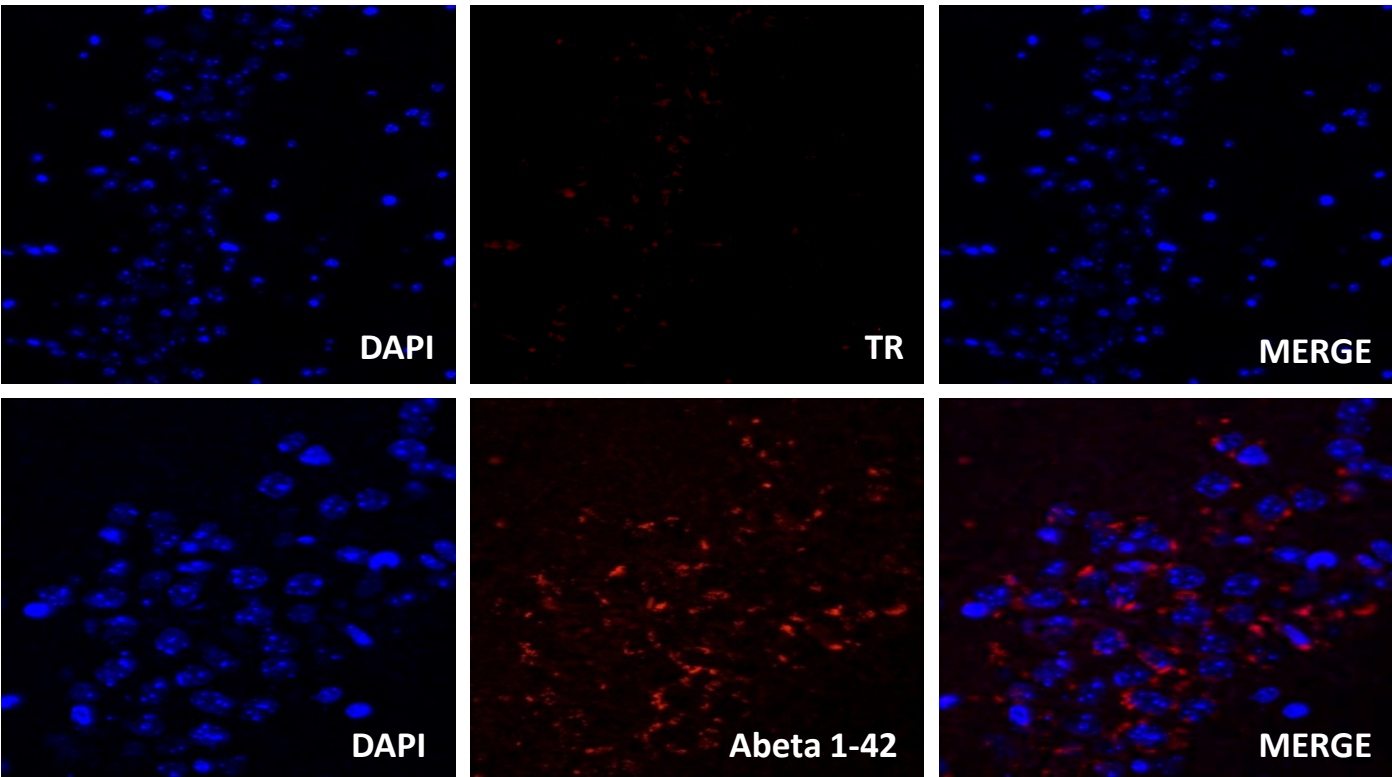

AD, anti-TNFSF10

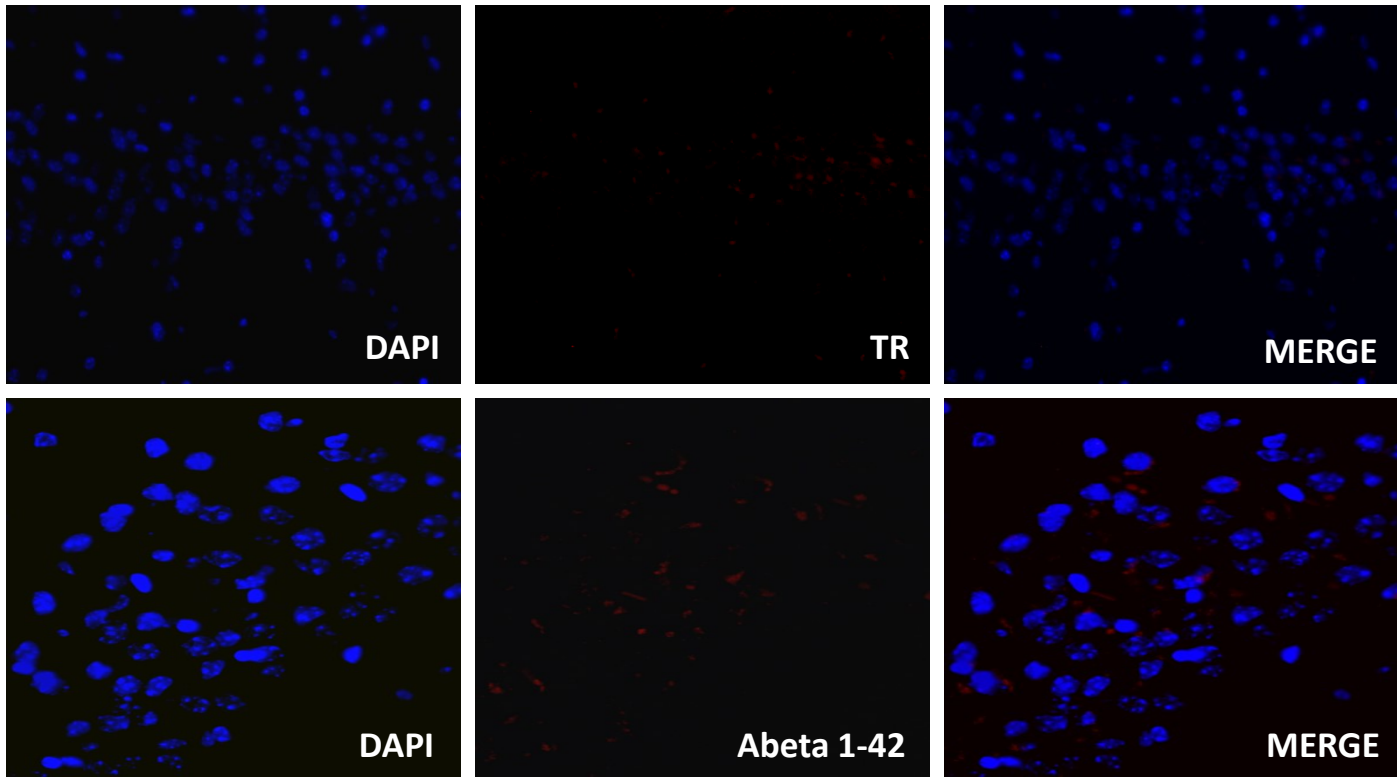

Supplement: Supplementary file 6 — Figure S6. Negative controls for Fig. 8, panel a (Aβ1–42 expression). Negative controls are reported in all panels marked with acronyms of secondary antibodies labeled with, respectively, Texas Red (TR) (PDF 455 kb) [file 12974_2019_1554_MOESM6_ESM.pdf]

WT, vehicle

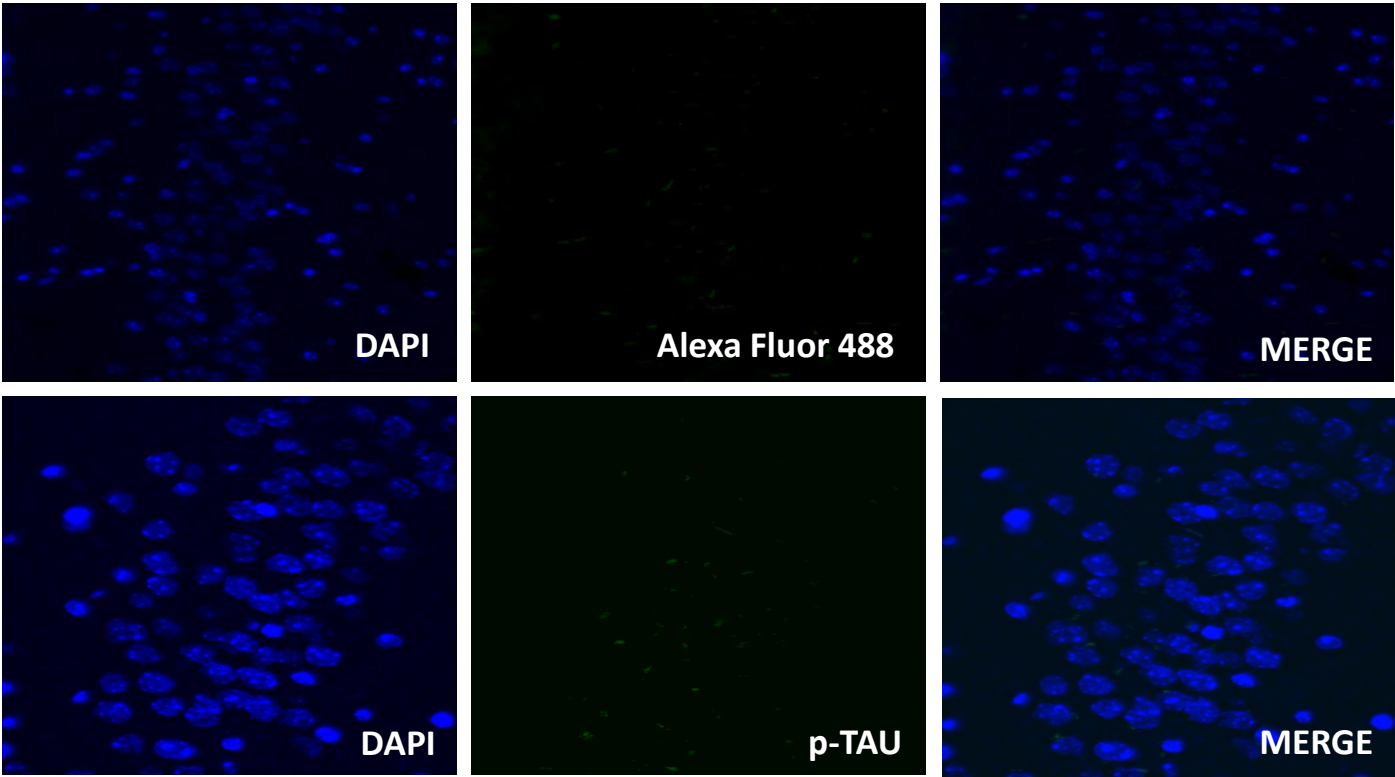

WT, anti-TNFSF10

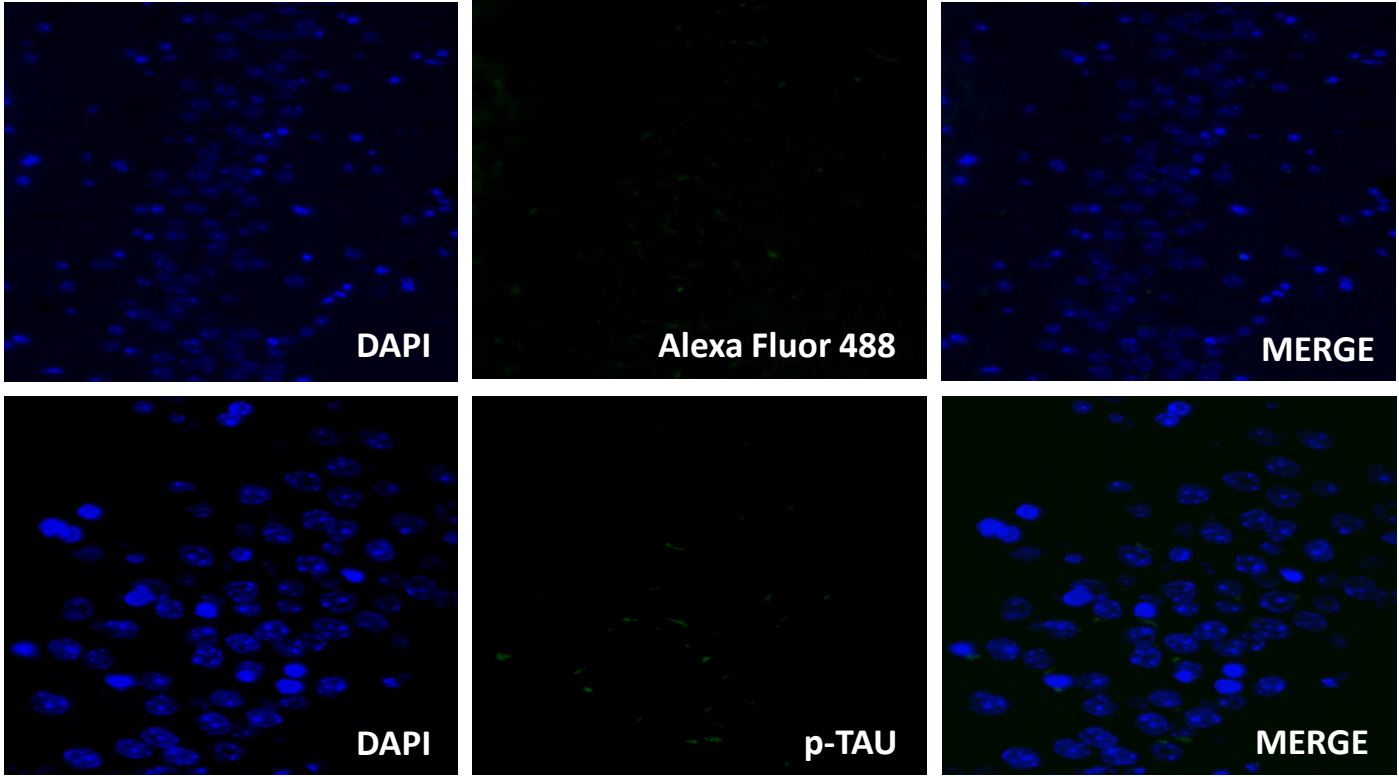

AD, vehicle

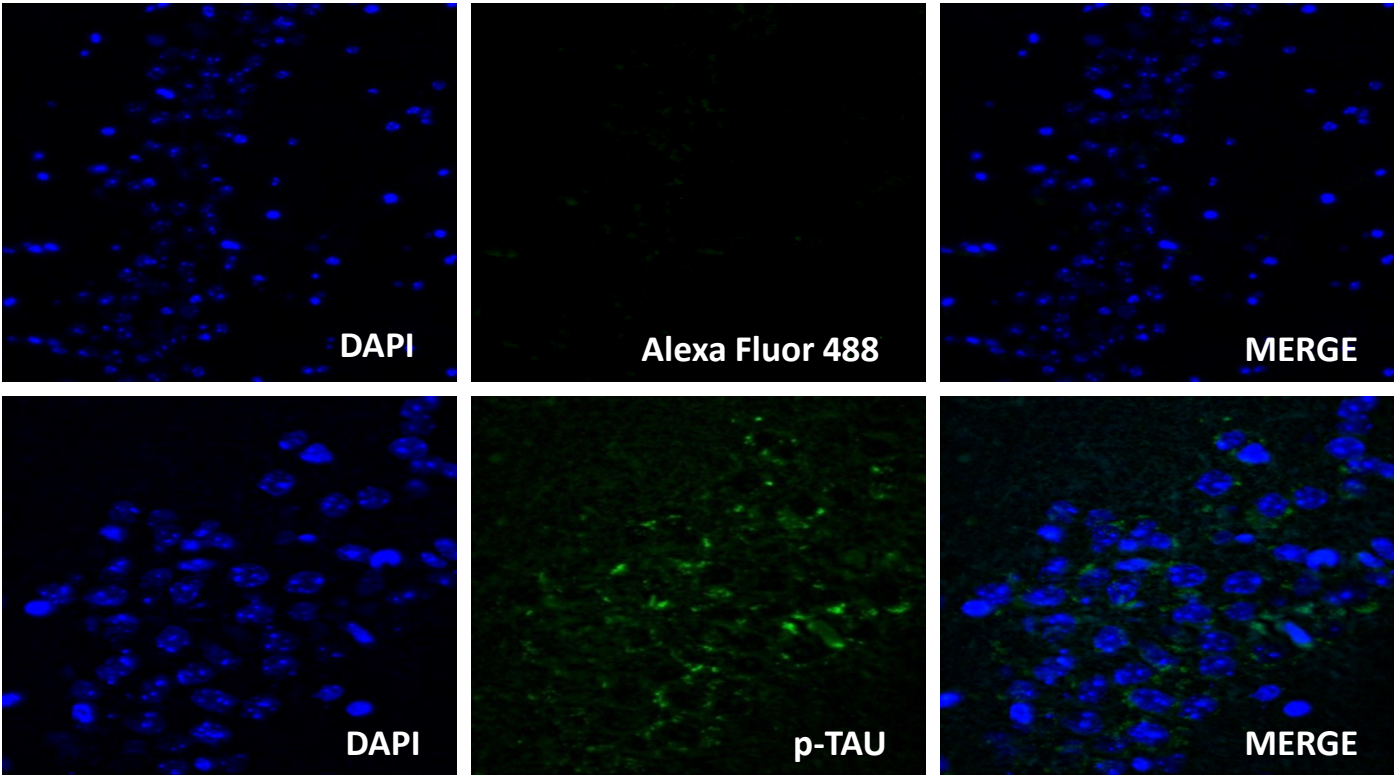

AD, anti-TNFSF10

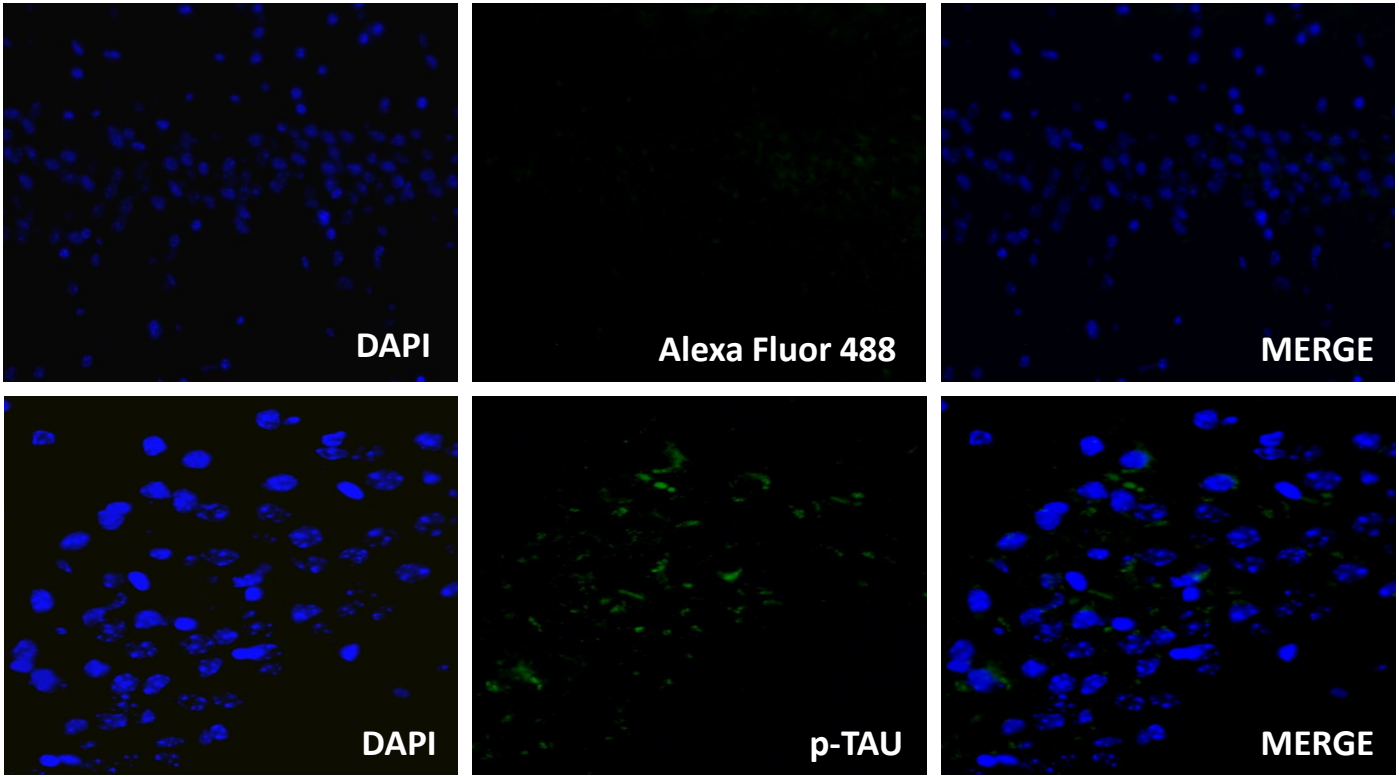

Supplement: Supplementary file 7 — Figure S7. Negative controls for Fig. 8, panel b (phosphorylated Tau protein expression). Negative controls are reported in all panels marked with acronyms of secondary antibodies labeled with, Alexa Fluor 488. (PDF 491 kb) [file 12974_2019_1554_MOESM7_ESM.pdf]
