# Supplementary material for: Beneficial effects of curtailing immune susceptibility in an Alzheimer’s disease model
Source: J Neuroinflammation. 2019 Aug 13;16:166. doi: 10.1186/s12974-019-1554-9 (PMC6693231; doi:10.1186/s12974-019-1554-9)
Supplement: Supplementary file 8 — Table S1. List of all antibodies used, with respective working dilutions for either WB or IHF, as well as Companies of origin and catalog number specification. (PDF 290 kb) [file 12974_2019_1554_MOESM8_ESM.pdf]

Antibody table

Antibodies	Diluition for WB	Diluition for IHF	Company (Catalog#)
Rabbit polyclonal Anti-TNFRSF10B	1:200	-	Abcam (ab8416)
Rabbit polyclonal Anti-TNFSF10	1:200	-	Abcam (ab2435)
Goat polyclonal Anti-GITR	1:1000	-	R&D Systems (AF524)
Rat monoclonal Anti-FOXP3	1:1000	1:500	eBioscience (13-5773)
Rabbit polyclonal Anti-IL-10	1:500	1:250	Abbiotec (250713)
Mouse monoclonal Anti-COX-2	1:500	-	BD Biosciences (610204)
Rabbit polyclonal Anti-NOS-2	1:500	-	Santa Cruz Biotechnology (sc-651)
Goat polyclonal Anti-IL-1β	1:500	-	R&D Systems (AF-401-NA)
Rabbit polyclonal Anti-TNF-α	1:1000	1:250	Abbiotec (251900)
Mouse monoclonal Anti-β-actin	1:1000	-	Santa Cruz Biotechnology (sc-47778)
Mouse monoclonal Anti-β-tubulin	1:1000	-	Santa Cruz Biotechnology (sc-5274)
Rabbit monoclonal Anti-CD3	-	1:200	Abcam (ab16669)
Mouse monoclonal Anti-GITR	-	1:500	Santa Cruz Biotechnology (sc-53972)
Rabbit polyclonal Anti-beta-amyloid 1-42	1:1000	1:500	Merck Millipore (AB5078P)
Goat polyclonal Anti-p-TAU	-	1:500	Santa Cruz Biotechnology (sc-16923)
Rat monoclonal Anti-CD11b	-	1:500	Serotec (MCA74G)
Rabbit IgG HRP Linked Whole Ab	1:10000	-	GE Healthcare (GENA934)
Mouse IgG HRP Linked Whole Ab	1:10000	-	GE Healthcare (GENA931)
Goat anti-mouse IgG-TR	-	1:500	Santa Cruz Biotechnology (sc-2781)
Goat anti-rat IgG - FITC	-	1:250	Merck Millipore (AP136F)
Goat anti-rabbit IgG-TR	-	1:500	Santa Cruz Biotechnology (sc-2780)
Alexa Fluor 488 goat anti-rabbit IgG	-	1:500	Life Technologies (A11008)
Alexa Fluor 488 donkey anti-goat IgG	-	1:500	Life Technologies (A32814)
